# Atomically Dispersed ZnN_4_ Sites Anchored on P‐Functionalized Carbon with Hierarchically Ordered Porous Structures for Boosted Electroreduction of CO_2_

**DOI:** 10.1002/advs.202306095

**Published:** 2023-12-07

**Authors:** Chenghong Hu, Wen Yao, Xianfeng Yang, Kui Shen, Liyu Chen, Yingwei Li

**Affiliations:** ^1^ Guangdong Provincial Key Laboratory of Fuel Cell Technology School of Chemistry and Chemical Engineering South China University of Technology Guangzhou 510640 P. R. China; ^2^ Analytical and Testing Centre South China University of Technology Guangzhou 510640 P. R. China

**Keywords:** accessibility, electrochemical CO_2_ reduction, electronic modulation, hierarchically ordered pores, single atom catalysts

## Abstract

Tuning the coordination structures of metal sites is intensively studied to improve the performances of single‐atom site catalysts (SASC). However, the pore structure of SASC, which is highly related to the accessibility of active sites, has received little attention. In this work, single‐atom ZnN_4_ sites embedded in P‐functionalized carbon with hollow‐wall and 3D ordered macroporous structure (denoted as H‐3DOM‐ZnN_4_/P‐C) are constructed. The creation of hollow walls in ordered macroporous structures can largely increase the external surface area to expose more active sites. The introduction of adjacent P atoms can optimize the electronic structure of ZnN_4_ sites through long‐rang regulation to enhance the intrinsic activity and selectivity. In the electrochemical CO_2_ reduction reaction, H‐3DOM‐ZnN_4_/P‐C exhibits high CO Faradaic efficiency over 90% in a wide potential window (500 mV) and a large turnover frequency up to 7.8 × 10^4^ h^−1^ at −1.0 V versus reversible hydrogen electrode, much higher than its counterparts without the hierarchically ordered structure or P‐functionalization.

## Graphical abstract

Single‐atomic ZnN_4_ sites are embedded in P‐functionalized carbon with unique hollow‐wall and 3D‐ordered macroporous structure, which display high accessibility and high intrinsic activity of active sites and thus ultra‐high activity and selectivity in electroreduction of CO_2_.

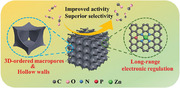

## 1 Introduction

Electrochemical CO_2_ reduction reaction (CO_2_RR) can convert CO_2_ to value‐added chemical feedstocks using renewable energy.^[^ ^1^ ^]^ In this concern, CO_2_RR to CO has proven to be a highly promising research target for industrial production, owing to its high selectivity, facile product separation from electrolytes, and the potential utility of CO as raw material for chemical synthesis.^[^ ^2^ ^]^ Transition‐metal single‐atom sites anchored on carbon supports, with high atom utilization efficiency and adjustable electronic structure, have emerged as potential electrocatalysts for CO_2_RR.^[^ ^3^ ^]^ For such catalysts, the carbon supports are vital to facilitate the accessibility and tune the electronic structure of metal sites.^[^ ^4^ ^]^

Metal–organic frameworks (MOFs), featured with high surface area and easy functionality, have been demonstrated to be excellent precursors for the synthesis of carbon‐supported transition‐metal single site catalysts.^[^ ^5^ ^]^ Especially, N‐rich zeolitic imidazolate frameworks (ZIFs) are intensively used to fabricate N‐coordinated metal sites embedded in carbon (MN_x_/C).^[^ ^6^ ^]^ Up to date, intensive studies have been focused on tuning the coordination structures of single metal sites to enhance their binding strength with the key intermediate (^*^COOH) in CO_2_RR for improved intrinsic activity.^[^ ^3^, ^7^ ^]^ However, the pore structure of the carbon supports, which is greatly related to the accessibility of active sites, is often overlooked. For most ZIF‐derived MN_x_/C, the MN_x_ sites are buried in microporous carbon skeletons, with only a small fraction of metal sites accessible in catalytic process.^[^ ^8^ ^]^ Although some templating or etching methods have been developed to construct hierarchically porous structures, the afforded macro‐ and meso‐pores usually suffered from disordering or inhomogeneity in multi‐scales.^[^ ^9^ ^]^ Very recently, polystyrene sphere‐templated method has been developed to synthesize ZIF‐derived MN_x_/C materials with ordered macroporous structures, whereas most of the active sites are still buried in the thick microporous pore walls.^[^ ^10^ ^]^ Therefore, it is highly desirable to develop hierarchically ordered macro‐ and mesoporous MN_x_/C with enhanced accessibility of MN_x_ sites for boosting the CO_2_RR efficiency, albeit challenging.

Herein, we report pore and surface engineering of carbon supports to regulate both the accessibility and intrinsic activity of MN_x_ sites for high‐efficiency electrochemical CO_2_RR. We successfully fabricate atomically dispersed ZnN_4_ sites embedded in P‐functionalized carbon with hollow‐wall and 3D ordered macroporous structure (denoted as H‐3DOM‐ZnN_4_/P‐C). In the synthesis, phytic acid (PA) is employed as an etching and functional agent to simultaneously create hollow‐wall structure and modify the external surface of ordered macroporous MOF. In the derived H‐3DOM‐ZnN_4_/P‐C, the hollow‐wall and ordered macroporous structure can greatly increase the external surface area to maximize the exposure of active sites. The introduced P atoms can tune the electronic structure of ZnN_4_ sites to optimize the adsorption strength of the key intermediate ^*^COOH in CO_2_RR. H‐3DOM‐ZnN_4_/P‐C exhibit high CO Faradaic efficiency (FE_CO_) over 90% in a wide potential window range from −0.4 to −0.9 V versus reversible hydrogen electrode (vs RHE) and a large turnover frequency (TOF) up to 7.8 × 10^4^ h^−1^ at −1.0 V. Further, a designed Zn‐CO_2_ battery with H‐3DOM‐ZnN_4_/P‐C cathode delivers a peak power density of 5.4 mW cm^−2^, and maintains a super stable charge‐discharge performance over 100 h. H‐3DOM‐ZnN_4_/P‐C was used as both anode and cathode materials in a dual‐electrode system of CO_2_RR coupled with hydrazine oxidation reaction (HzOR). This system can greatly lower the cell voltage by 0.71 V at a current density of 10 mA cm^−2^ than that of traditional CO_2_RR coupled with an oxygen evolution reaction (OER) system, theoretically saving 38% of energy consumption.

## 2 Results and Discussion

**Figure 1:**
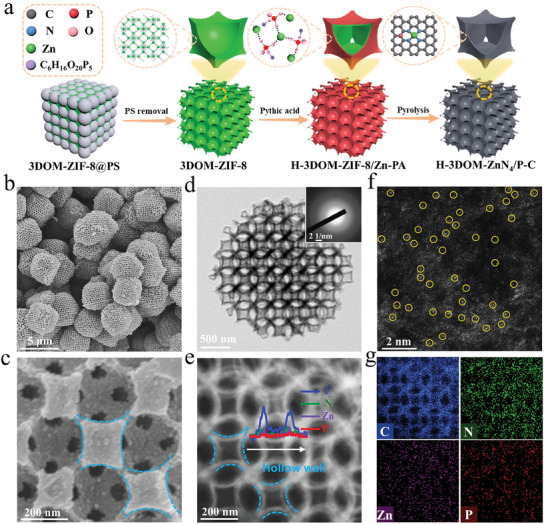
a) Schematic illustration of the synthesis of H‐3DOM‐ZnN_4_/P‐C. b,c) SEM images of H‐3DOM‐ZnN_4_/P‐C. d) TEM image of H‐3DOM‐ZnN_4_/P‐C. e) HAADF‐STEM image of H‐3DOM‐ZnN_4_/P‐C. f) Aberration‐corrected HAADF‐STEM image of H‐3DOM‐ZnN_4_/P‐C displays the Zn single atoms (highlighted by yellow circles) are dispersed on the carbon supports. g) EDS element mappings of H‐3DOM‐ZnN_4_/P‐C.

The preparation of H‐3DOM‐ZnN_4_/P‐C is illustrated in **Figure** **1**a. First, 3DOM‐ZIF‐8 was synthesized using a polystyrene sphere monolith template with a diameter size of ≈410 nm (Figure 1a; Figure S1, Supporting Information).^[^ ^11^ ^]^ The powder X‐ray diffraction (XRD) patterns of 3DOM‐ZIF‐8 are consistent with the simulated ZIF‐8 (Figure S2, Supporting Information). The scanning electron microscopy (SEM) images of 3DOM‐ZIF‐8 display a uniform cubic morphology and 3D‐ordered macroporous structure (Figure S3, Supporting Information). The transmission electron microscope (TEM) images of 3DOM‐ZIF‐8 confirm the periodic arrangement of macropores (Figure S3, Supporting Information). Moreover, the pore wall of 3DOM‐ZIF‐8 displays a solid character.

Subsequently, 3DOM‐ZIF‐8 was immersed in PA‐methanol solution for selective etching to afford ZIF‐8 coated with PA cross‐linked Zn complex (H‐3DOM‐ZIF‐8/Zn‐PA). H‐3DOM‐ZIF‐8/Zn‐PA retains the cubic morphology and ordered macroporous structure of 3DOM‐ZIF‐8 (Figure S4, Supporting Information). However, the macropore walls of H‐3DOM‐ZIF‐8/Zn‐PA turn to be hollow, as observed from the TEM images (Figure S4, Supporting Information). The XRD patterns of H‐3DOM‐ZIF‐8/Zn‐PA show similar characteristic peaks assigned to the pure phase of 3DOM‐ZIF‐8 (Figure S2, Supporting Information), suggesting a part of ZIF‐8 is preserved after the etching treatment. The Fourier transform infrared (FTIR) spectrum of H‐3DOM‐ZIF‐8/Zn‐PA displays peaks attributed to the stretching vibration of functional groups from both ZIF‐8 and Zn‐PA (Figure S5, Supporting Information), indicating the partial substitution of 2‐methylimidazole by PA and the formation of Zn‐PA. The Zeta potentials change from positive to negative after PA etching (Figure S6, Supporting Information), implying that Zn‐PA is attached to the surface of 3DOM‐ZIF‐8. The water contact angle measurement shows that H‐3DOM‐ZIF‐8/Zn‐PA possesses a smaller contact angle of water compared to 3DOM‐ZIF‐8 (Figure S7, Supporting Information), suggesting the coating of more hydrophilic Zn‐PA on 3DOM‐ZIF‐8. The etching mechanism was then studied. The molecular size of PA (1.28 nm × 1.16 nm × 0.93 nm) is larger than the pore size of 3DOM‐ZIF‐8 (0.34 nm), eliminating the possibility of competitive coordination between Zn‐imidazole and Zn‐PA. The pH value of the methanol solution of PA is measured to be 1.6 (Table S1, Supporting Information), implying that PA in methanol would release protons. Therefore, the etching of MOFs should be originated from the release of H^+^ from PA. Based on the above results, the formation process of the hollow walls is illustrated. The proton H^+^ in the PA‐methanol solution can diffuse into the microporous walls of 3DOM‐ZIF‐8, while the remaining PA ions with a larger size than the micropore window remain on the external surface. The diffused proton H^+^ causes the break of the Zn‐imidazole coordination. The dissociated Zn ions diffuse outside and then coordinate with PA ions to form Zn‐PA on the macropore surface. As the outer layer of ZIF‐8 is more chemically robust than the inner layer,^[^ ^12^ ^]^ the interior is preferentially etched by PA, resulting in a solid‐to‐hollow transformation of the macropore wall.

Finally, H‐3DOM‐ZIF‐8/Zn‐PA was calcined at 950 °C in an argon atmosphere to obtain H‐3DOM‐ZnN_4_/P‐C. The XRD patterns of H‐3DOM‐ZnN_4_/P‐C show only two broad peaks at ≈23.5^o^ and 44.1^o^, assigned to the (002) and (100) planes of carbon matrix, respectively (Figure S8, Supporting Information).^[^ ^13^ ^]^ H‐3DOM‐ZnN_4_/P‐C retains the ordered macroporous and hollow‐wall structure after pyrolysis (Figure 1b–d). The macropore size is measured to be 382 nm. The high‐angle annular dark field scanning transmission electron microscopy (HAADF‐STEM) image combined with the line scanning profiles manifest the preservation of the hollow‐wall structure (Figure 1e). The selected area electron diffraction (SAED) image of H‐3DOM‐ZnN_4_/P‐C shows diffraction rings assigned to carbon without crystallized Zn species (insert in Figure 1d). The aberration‐corrected HAADF‐STEM image elucidates that Zn atoms are atomically dispersed on the carbon skeleton without the observation of Zn clusters or nanoparticles (Figure 1f). The element mappings of H‐3DOM‐ZnN_4_/P‐C demonstrate that Zn, P, and N are distributed evenly on the carbon substrate (Figure 1g). The content of Zn is measured to be 1.56 wt.% by atomic absorption spectroscopy (AAS) (Table S2, Supporting Information).

**Figure 2:**
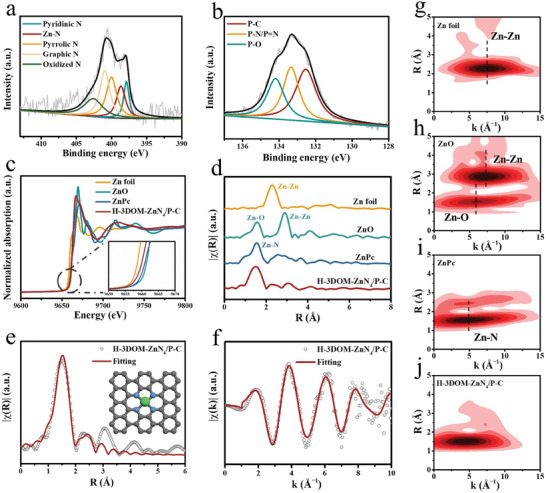
a) High‐resolution N 1s and b) P 2p XPS spectra of H‐3DOM‐ZnN_4_/P‐C. c) Zn K‐edge XANES spectra of H‐3DOM‐ZnN_4_/P‐C with a zoomed‐in view in the inset. d) FT‐EXAFS spectra of H‐3DOM‐ZnN_4_/P‐C, Zn foil, ZnO, and ZnPc at Zn K‐edge. EXAFS fitting analysis of H‐3DOM‐ZnN_4_/P‐C in e) R space (the inset is the coordination structure of Zn) and f) k space. EXAFS wavelet transforms of g) Zn foil, h) ZnO, i) ZnPc, and j) H‐3DOM‐ZnN_4_/P‐C.

The elemental chemical states of H‐3DOM‐ZnN_4_/P‐C are revealed by X‐ray photoelectron spectroscopy (XPS). The C 1s XPS spectra display four peaks at 284.5, 285.2, 286.2, and 288.4 eV (Figure S9, Supporting Information), corresponding to graphitic sp^2^ carbon (C═C), carbon coordinated with P or N (C─P or C─N), and O═C─O bonds, respectively.^[^ ^13b^ ^]^ H‐3DOM‐ZnN_4_/P‐C exhibits Zn 2p_3/2_ at 1021.7 eV (Figure S10, Supporting Information), which is between 1022.8 eV (Zn^2+^) and 1021.2 eV (Zn^0^), indicating that the valence state of Zn is between +2 and 0.^[^ ^14^ ^]^ The N 1s spectra display five deconvoluted components at 397.8, 398.6, 400.1, 401.0, and 402.6 eV (**Figure** **2**a), characteristic of pyridinic N, Zn─N, pyrrolic N, graphitic N, and oxidized N, respectively.^[^ ^11b^ ^]^ For the P 2p XPS spectra of H‐3DOM‐ZnN_4_/P‐C, the characteristic peaks at 132.5, 133.3, and 134.2 eV are corresponding to the P─C, P═N/P─N and P─O bonds, respectively (Figure 2b).^[^ ^15^ ^]^ The observed Zn─N bonding and absence of Zn─P bonding suggest that N atoms are coordinated to the atomically dispersed Zn, while P is incorporated in the carbon skeleton without coordinating with Zn.

To further determine the coordination structure of Zn in H‐3DOM‐ZnN_4_/P‐C, X‐ray absorption fine structure measurement was conducted. The Zn K‐edge X‐ray absorption near edge structure (XANES) spectra show that the absorption edge of H‐3DOM‐ZnN_4_/P‐C is situated between ZnO and Zn foil (Figure 2c), which confirms that the Zn valence state in H‐3DOM‐ZnN_4_/P‐C is between +2 and 0. It is worth noting that the absorption edge of H‐3DOM‐ZnN_4_/P‐C shifts to lower energy compared with that of Zn phthalocyanine (ZnPc), implying the enhancement of local charge density of Zn sites by the introduction of P atoms. The extended X‐ray absorption fine structure (EXAFS) data were used to quantify coordination numbers and bond lengths. The Fourier‐transformed (FT) k^3^‐weighted EXAFS spectra of H‐3DOM‐ZnN_4_/P‐C present a prominent peak at 1.53 Å (Figure 2d), which can be ascribed to the Zn─N coordination. No Zn─Zn interaction (2.24 Å) is detected in H‐3DOM‐ZnN_4_/P‐C, eliminating the existence of Zn aggregates. Wavelet transform of Zn─K edge EXAFS oscillations of H‐3DOM‐ZnN_4_/P‐C confirm the sole existence of Zn─N coordination (Figure 2g–j). EXAFS fitting was then performed to obtain the quantitative local structure parameters of the Zn atom. The fitting spectra of H‐3DOM‐ZnN_4_/P‐C in R and k space suggest that the Zn atom is coordinated by four N atoms (Figure 2e,f; Figure S11–S13, and Table S3, Supporting Information). The above results imply that the P atom is doped in the carbon matrix without coordination with Zn (inset in Figure 2e). The coordinated N atoms can well stabilize Zn atoms, and the P atoms in the surrounding carbon sphere can tune the electronic structure of Zn sites.

Next, we prepared several control samples including conventional ZnN_4_ embedded in P‐doped carbon with microporous structure (ZnN_4_/P‐C), ZnN_4_ embedded in P‐doped carbon with 3DOM structure (3DOM‐ZnN_4_/P‐C) to verify the role of the hollow wall structure, and ZnN_4_ embedded in carbon with 3DOM structure (3DOM‐ZnN_4_/C) to identify the effect of P doping. The XRD patterns of all samples show similar broad peaks assigned to carbon (Figure S8, Supporting Information). Raman spectra of 3DOM‐ZnN_4_/P‐C suggest more structural defects in the carbon network due to the P doping as compared to 3DOM‐ZnN_4_/C (Figure S14, Supporting Information). The Zn content in the samples is similar, as measured by AAS (Table S2, Supporting Information). The SEM and TEM images of ZnN_4_/P‐C display conventional dodecahedral morphology and microporous structure (Figure S15, Supporting Information), while 3DOM‐ZnN_4_/P‐C (Figure S16, Supporting Information) and 3DOM‐ZnN_4_/C (Figure S17, Supporting Information) both show similar 3DOM and solid‐wall structures. The Zn 2p_3/2_ XPS peak of 3DOM‐ZnN_4_/P‐C is located at 1021.8 eV, which shifts to lower binding energy compared with that of 3DOM‐ZnN_4_/C (1021.9 eV) (Figure S10, Supporting Information). The increased charge density of Zn after P doping suggests the existence of electronic interaction between the Zn and P atom, consistent with the XANES analysis.

**Figure 3:**
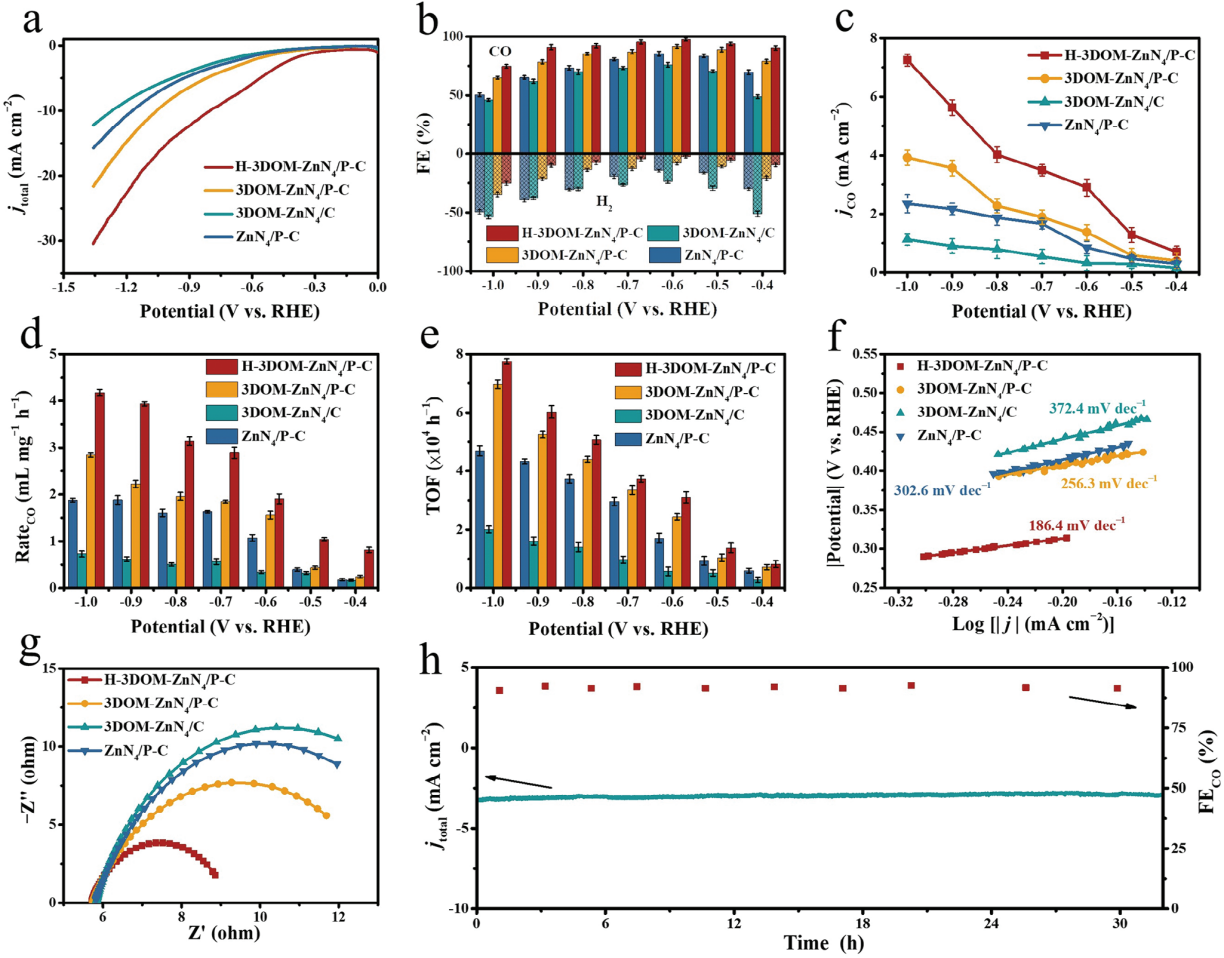
a) LSV curves, b) product distribution, c) CO partial current densities, d) CO formation rate, e) normalized TOF, f) Tafel slop, and g) Nyquist plots for H‐3DOM‐ZnN_4_/P‐C, 3DOM‐ZnN_4_/P‐C, 3DOM‐ZnN_4_/C, and ZnN_4_/P‐C in 0.1 m CO_2_‐saturated KHCO_3_. h) Amperometric *i*−*t* stability of H‐3DOM‐ZnN_4_/P‐C at −0.6 V versus RHE.

The CO_2_ electroreduction performance of the as‐obtained catalysts was evaluated in CO_2_‐saturated 0.1 m KHCO_3_ electrolytes using an H‐type cell with a three‐electrode system. All catalysts show a higher current density in CO_2_‐saturated electrolytes compared with N_2_‐saturated electrolytes, indicating a preference for CO_2_RR rather than hydrogen evolution reaction (HER) under the CO_2_‐saturated condition (Figure S18, Supporting Information). As revealed by the linear sweep voltammetry (LSV) curves (**Figure** **3**a), H‐3DOM‐ZnN_4_/P‐C exhibits larger current densities than 3DOM‐ZnN_4_/P‐C, 3DOM‐ZnN_4_/C, and microporous ZnN_4_/P‐C, indicating the introduction of P atom and the construction of H‐3DOM structures can both promote the electrochemical reduction performance.

We then carried out chronopotentiometry measurements in the range of −0.4 to −1.0 V (vs RHE, same below if not mentioned) to quantitatively determine the products (Figure 3b). Only CO and H_2_ could be detected by gas chromatography, while no liquid products were present in ^1^H nuclear magnetic resonance (Figure S19, Supporting Information). The catalysts with P doping exhibit higher CO selectivity than P‐free catalysts. The FE_CO_ at the H‐3DOM‐ZnN_4_/P‐C electrode is above 90% at a wide potential window range from −0.4 to −0.9 V with a maximum FE_CO_ of ≈100% at −0.6 V, which outperforms most of the reported single site catalysts (Table S4, Supporting Information). Meanwhile, the partial current density of CO (*j*_CO_) on H‐3DOM‐ZnN_4_/P‐C is higher than 3DOM‐ZnN_4_/P‐C, 3DOM‐ZnN_4_/C, and ZnN_4_/P‐C in the controlled potential range (Figure 3c). The CO production rate over H‐3DOM‐ZnN_4_/P‐C reaches 4.0 mL mg_catalyst_^−1^ h^−1^ at −0.9 V, which is 1.8, 2.2, and 6.7 times that of 3DOM‐ZnN_4_/P‐C (2.2 mg_catalyst_^−1^ h^−1^), ZnN_4_/P‐C (1.8 mg_catalyst_^−1^ h^−1^), and 3DOM‐ZnN_4_/C (0.6 mg_catalyst_^−1^ h^−1^), respectively (Figure 3d). The above results indicate the construction of the hollow‐wall and 3DOM structures and the introduction of the P atom could enhance the conversion of CO_2_ to CO.

As shown in Figure 3h, the time dependence of FE_CO_ and current density for H‐3DOM‐ZnN_4_/P‐C reveal that the CO_2_RR performance remains almost unchanged during continuous operation at −0.6 V over 30 h. Furthermore, H‐3DOM‐ZnN_4_/P‐C can also maintain the higher current density of 7.5 mA cm^−2^ at −0.9 V and FE_CO_ above 90% over 36 h (Figure S20, Supporting Information). The XRD patterns of H‐3DOM‐ZnN_4_/P‐C after the reaction show only the diffraction peaks of carbon and no metal aggregates (Figure S21a, Supporting Information). SEM and TEM images show the maintenance of the 3D‐ordered macroporous and hollow‐wall structure after reaction (Figure S21b,c, Supporting Information). Aberration‐corrected HAADF‐STEM image shows the Zn atoms uniformly disperse on the carbon skeleton (Figure S21d, Supporting Information). The above results suggest the outstanding stability of H‐3DOM‐ZnN_4_/P‐C for CO_2_RR.

The Tafel slope of H‐3DOM‐ZnN_4_/P‐C, 3DOM‐ZnN_4_/P‐C, ZnN_4_/P‐C, and 3DOM‐ZnN_4_/C is determined to be 186.4, 256.3, 302.6, and 372.4 mV dec^−1^ (Figure 3f), respectively. The calculated Tafel slopes suggest that the first electron transfer to generate ^*^COOH intermediate is the rate‐determining step in CO_2_RR. The reduced Tafel slope of H‐3DOM‐ZnN_4_/P‐C implies accelerated kinetics for ^*^COOH formation due to the hollow‐wall structure and P doping.^[^ ^14^, ^16^ ^]^ Nyquist plot for electrochemical impedance spectroscopy (EIS) spectra of H‐3DOM‐ZnN_4_/P‐C exhibits the lowest interfacial charge transfer resistance among the prepared catalysts (Figure 3g), indicating the highest charge transfer kinetics of H‐3DOM‐ZnN_4_/P‐C.^[^ ^17^ ^]^

**Figure 4:**
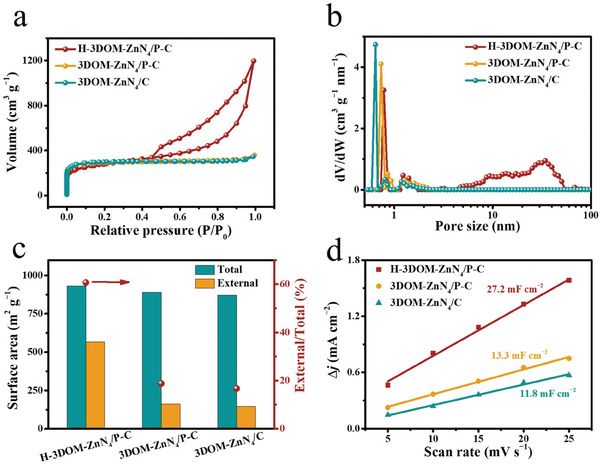
a) N_2_ adsorption–desorption isotherms, b) pore size distribution calculated from the non‐local density functional theory model, c) specific external surface areas and total surface area for H‐3DOM‐ZnN_4_/P‐C, 3DOM‐ZnN_4_/P‐C, and 3DOM‐ZnN_4_/C, respectively. d) ECSA of the H‐3DOM‐ZnN_4_/P‐C, 3DOM‐ZnN_4_/P‐C, and 3DOM‐ZnN_4_/C in 0.1 m CO_2_‐saturated KHCO_3_.

The origin of the high activity and selectivity of H‐3DOM‐ZnN_4_/P‐C was further investigated. The N_2_ sorption isotherm of H‐3DOM‐ZnN_4_/P‐C shows a typical adsorption curve of type I plus IV, indicating the presence of mico‐ and mesopores (**Figure** **4**a).^[^ ^18^ ^]^ Comparably, 3DOM‐ZnN_4_/P‐C and 3DOM‐ZnN_4_/C exhibit dominant microporous structures, which is confirmed by the pore size distribution curve (Figure 4b). All the materials exhibit considerable total surface areas, whereas H‐3DOM‐ZnN_4_/P‐C possesses the largest external surface area (565.8 cm^2^ g^−1^), which is 3.5, and 4.0 times that of 3DOM‐ZnN_4_/P‐C (160.6 cm^2^ g^−1^), and 3DOM‐ZnN_4_/C (140.5 cm^2^ g^−1^), respectively (Figure 4c). Considering that the electrocatalytic reaction mainly proceeds on the external surface of catalysts, H‐3DOM‐ZnN_4_/P‐C with a larger external surface area will provide more accessible active sites. The electrochemical active surface areas (ECSA) were deduced by measuring the corresponding double‐layer capacitances (*C*_dl_) by cyclic voltammetry (CV) and EIS methods (Figure 4d; Figure S22 and Table S5, Supporting Information). H‐3DOM‐ZnN_4_/P‐C has a larger *C*_dl_ value (27.2 mF cm^−2^) than 3DOM‐ZnN_4_/P‐C (13.3 mF cm^−2^), and 3DOM‐ZnN_4_/C (11.8 mF cm^−2^) according to CV method measured in the nonfaradaic region, which is consistent with the values measured by EIS methods in the faradaic region. These results confirm that H‐3DOM‐ZnN_4_/P‐C possesses the largest accessible electrochemical active sites. The above results demonstrate that the construction of H‐3DOM structures can expose more electrochemically accessible active sites.

The critical role of Zn sites in the catalytic reaction was further verified by thiocyanide (SCN^−^) poisoning as SCN^−^ can strongly bind to the Zn sites to block their adsorption of reaction substrates (Figure S23, Supporting Information). After the introduction of SCN^−^ (0.1 m) into the reaction system, the current density and FE_CO_ decrease significantly, confirming that that Zn sites should be the active centers for CO_2_RR. To reveal the intrinsic activity of active Zn sites in the catalysts, TOF values were calculated based on the number of accessible active Zn sites (Figure 3e). H‐3DOM‐ZnN_4_/P‐C shows a TOF value of 7.8 × 10^4^ h^−1^ at −1.0 V, which is similar to that of 3DOM‐ZnN_4_/P‐C but significantly higher than that of 3DOM‐ZnN_4_/C, suggesting that the introduction of P atom can enhance the intrinsic activity of Zn sites. It is worth noting that H‐3DOM‐ZnN_4_/P‐C shows a higher TOF value than most of the reported single‐site catalysts (Table S4, Supporting Information).

The effect of calcination temperatures on the catalyst performance was investigated. The Zn contents of the catalysts are decreased with the increase of calcination temperatures (Table S6, Supporting Information). The calcination temperature increased to 1050 °C or decreased to 850 °C leads to lower current density and FE_CO_ of the catalysts compared to 3DOM‐ZnN_4_/P‐C obtain at 950 °C (Figure S24, Supporting Information). The decrease of calcination temperature could not form a porphyrin‐like ZnN_4_ plane thus lower intrinsic activity.^[^ ^19^ ^]^ The increase of calcination temperature to 1050 °C would greatly reduce the Zn content thus reduce the activity. Therefore, it is important to optimize the calcination temperature to balance the densities and intrinsic activities of Zn sites.

We further studied the effect of PA on the activity of ZnN_4_ sites. The catalyst obtained using 100 mg of PA has the highest current density and FE_CO_ (Figure S25, Supporting Information). The use of lower content of PA (50 mg) cannot construct the hollow‐wall structure and provide sufficient P source to regulate the electronic structure of Zn site (Figure S26, Supporting Information). However, the use of too much PA (200 mg) leads to the collapse of the 3DOM porous structure.

To further reveal the promoting effect of P on ZnN_4_ sites, we performed density functional theory (DFT) calculation for CO_2_RR. Basic models based on the ZnN_4_ site with surrounding P atoms in different coordination shells were constructed. The positions of P in the second (ZnN_4_/P(II)), third (ZnN_4_/P(III)), and fourth coordination shell of the Zn site (ZnN_4_/P(IV)) were determined by using the most stable configuration with the lowest energy (Figure S27, Supporting Information).

**Figure 5:**
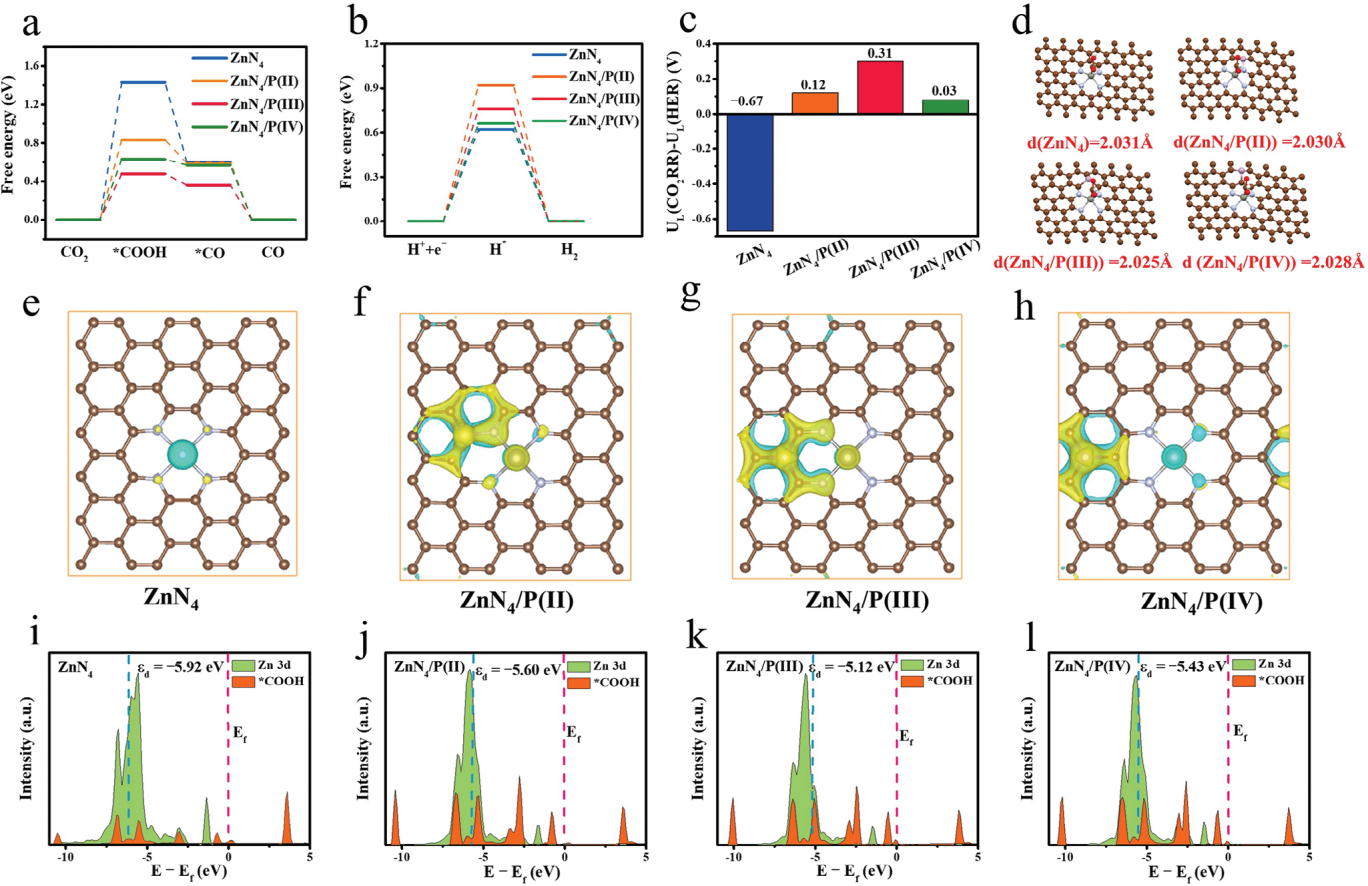
DFT studies on the CO_2_RR mechanism of different catalyst models. a) Free energy profiles for the optimized ZnN_4_, ZnN_4_/P(II), ZnN_4_/P(III) and ZnN_4_/P(IV) models during the CO_2_RR, and b) HER. c) Difference in limiting potentials for CO_2_RR and HER on different models. d) Zn−C bond distance with different models. Calculated charge density differences for e) ZnN_4_, f) ZnN_4_/P(II), g) ZnN_4_/P(III), and e) ZnN_4_/P(IV) models (yellow and cyan areas represent charge density increase and decrease, respectively). Projected DOS of i) ZnN_4_, j) ZnN_4_/P(II), k) ZnN_4_/P(III), and l) ZnN_4_/P(IV) models.

The electroreduction of CO_2_ to CO generally encompasses the following three elementary steps, i.e., 1) CO_2_ + ^*^ + H^+^ + e^−^ → ^*^COOH; 2) ^*^COOH + H^+^ + e^−^ → ^*^CO +H_2_O; and 3) ^*^CO → CO + ^*^. The adsorption configurations of CO_2_, intermediates, and CO on Zn sites of each model were optimized (Figure S28, Supporting Information). As shown in **Figure** **5**a, the formation of ^*^COOH species is endothermic in both models, which is regarded as the rate‐limiting step, consistent with the Tafel slope results. The Gibbs free energies (ΔG) for the formation of ^*^COOH are 1.43, 0.82, 0.48, and 0.62 eV on ZnN_4_, ZnN_4_/P(II), ZnN_4_/P(III), and ZnN_4_/P(IV), respectively. Therefore, the introduction of the P atom near the ZnN_4_ site can significantly reduce the ΔG for the formation of ^*^COOH, among which the P site located in the third coordination layer of the ZnN_4_ sites (ZnN_4_/P(III)) shows the minimized ΔG. The Gibbs free energies for the HER were further calculated (Figure S29, Supporting Information). As shown in Figure 5b, the introduction of P element increases ΔG for H_2_ formation, indicating that the HER process requires higher energy intake and is difficult to occur. The limiting potential difference (ΔU) between CO_2_RR and HER (ΔU = U_L_(CO_2_) − U_L_(H_2_), where U_L_ = ΔG_0_/e) is used as the indicator of the CO_2_RR selectivity, where a more positive ΔU value indicates a higher selectivity of CO_2_RR.^[^ ^20^ ^]^ As shown in Figure 5c, the ZnN_4_/P(III) site shows the most positive ΔU value (0.31 V) than the ZnN_4_ (−0.67 V), ZnN_4_/P(II) (0.12 V) and ZnN_4_/P(IV) (0.03 V) sites. These results imply the long‐range electronic regulation caused by the P atom endows the catalyst with excellent capability in CO_2_RR.

The charge density difference and projected density of states (DOS) were calculated to clarify the long‐range electronic regulation of the ZnN_4_ site by the adjacent P atom. As shown in Figure 5e, ZnN_4_ sites display symmetric charge distribution. When P atoms are introduced in different coordination shells of the Zn sites, the charge distribution is obviously redistributed, resulting in the change of oxidation states from +1.29 *e* (ZnN_4_) to +1.27 *e* (ZnN_4_/P(II)), +1.15 *e* (ZnN_4_/P(III)), and +1.17 *e* (ZnN_4_/P(IV)) (Table S7, Supporting Information). The DOS of Zn 3d, and ^*^COOH 2p orbitals were then used to study the strength variation between the metal center and the adsorbent. As observed from Figure 5i–l, the gap between the d‐band center (*ε*_d_) of Zn and the Fermi energy level (*E*_f_) is 5.92 eV in the ZnN_4_ configuration. When P atom is introduced near the ZnN_4_ sites, the gap is significantly narrowed to 5.60, 5.12, and 5.43 eV of ZnN_4_/P(II), ZnN_4_/P(III), and ZnN_4_/P(IV), respectively, indicating that the adsorption strength of ^*^COOH at the Zn center is enhanced. Moreover, the Zn─C bond between the Zn site and the adsorbed ^*^COOH intermediate is shortened after the introduction of P sites, confirming the enhanced binding strength of the metal center to the reaction intermediate (Figure 5d). Therefore, it can be concluded that the introduction of heteroatom P can optimize the electronic structure of active Zn center with increased local charge density, and thus enhance the binding strength of ^*^COOH, leading to superior CO_2_RR activity.

**Figure 6:**
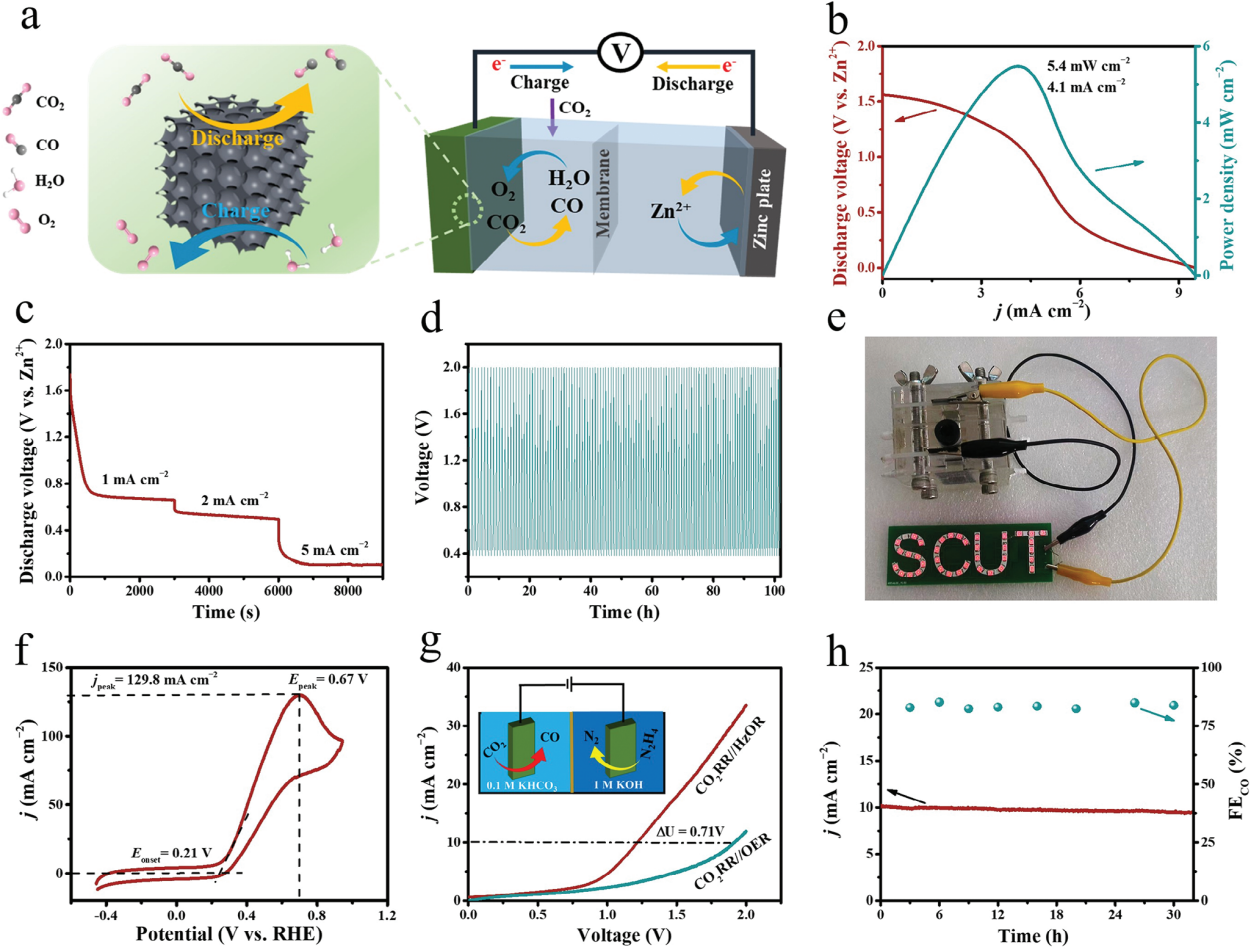
a) Schematic configuration of the aqueous ZCB with the H‐3DOM‐ZnN_4_/P‐C cathode and Zn plate anode. b) Polarization plot and power density. c) Galvanostatic discharge curves at several current densities. d) Galvanostatic discharge–charge cycling curves at 1 mA cm^−2^ for 100 h. e) Digital photograph of a LED illumed by the ZCB. f) CV curve of H‐3DOM‐ZnN_4_/P‐C in 1 m KOH with 0.1 m N_2_H_4_. g) The schematic configuration of the HzOR//CO_2_RR electrolyzer (inset) and polarization curves of the electrolyzer with and without 0.1 m hydrazine at a scan rate of 5 mV s^−1^. h) Cell current as a function of time recorded with H‐3DOM‐ZnN_4_/P‐C as both anodic and cathodic electrodes at a voltage of −1.2 V, and the corresponding FE_co_ from the chronopotentiometry measurement.

Inspired by the excellent performance of H‐3DOM‐ZnN_4_/P‐C for CO_2_RR, H‐3DOM‐ZnN_4_/P‐C was further employed as the cathode to assemble a rechargeable Zn‐CO_2_ battery (ZCB).^[^ ^21^ ^]^ In 0.8 m KOH + 0.02 m ZnOOCCH_3_ basic anodic electrolyte, the dissolution and precipitation of Zn anode are corresponding to the discharge and charge processes, respectively. In 0.8 m KHCO_3_ cathodic electrolyte, CO_2_RR and OER are corresponding to the discharge and charge processes, respectively. **Figure** **6**a shows the schematic configuration of a rechargeable ZCB based on H‐3DOM‐ZnN_4_/P‐C cathode. Figure 6b shows the discharge curve during the working of the ZCB. The calculated peak power density reaches 5.4 mW cm^−2^ at 4.1 mA cm^−2^. In addition, the voltage plateau corresponding to different current densities of 1, 2, and 5 mA cm^−2^ remains relatively stable (Figure 6c). Furthermore, no obvious change in the voltage on H‐3DOM‐ZnN_4_/P‐C‐based ZCB at 1 mA cm^−2^ is observed during the continuous charge and discharge operations for 100 h (Figure 6d), suggesting superior rechargeable durability. The ZCB based on the H‐3DOM‐ZnN_4_/P‐C cathode can light up all the red LED lights in parallel (Figure 6e), indicating the potential use of the developed ZCB in energy conversion devices.

We further set up a two‐electrode electrolyzer for CO_2_RR. For traditional CO_2_RR devices, the total energy efficiency is greatly limited by the kinetically sluggish and thermodynamically unfavorable anodic OER.^[^ ^22^ ^]^ We employed hzOR with much lower oxidation potential to replace anodic OER in the CO_2_RR systems. As shown in Figure 6f, the H‐3DOM‐ZnN_4_/P‐C shows the onset potential (*E*_onset_) of only 0.21 V and reaches the highest current density of 129.8 mA cm^−2^ at 0.67 V in HzOR, which is comparable favorably to most of the previously reported transition metal‐based electrocatalysts (Table S8, Supporting Information). The stability of the catalyst was investigated by CV curves before and after long‐term electrolysis. The catalytic activity of the catalyst after 48 h of reaction maintains to be 90% of the fresh one (Figure S30, Supporting Information), indicating that the material has superior stability in the alkaline solution. Then, H‐3DOM‐ZnN_4_/P‐C was used as both anode and cathode materials to assemble a dual‐electrode system coupled with CO_2_RR and HzOR to verify the feasibility of HzOR as a substitute for anodic OER reaction (Figure 6g). The effect of HzOR on cell voltage reduction is verified by comparing polarization curves of CO_2_RR//HzOR with CO_2_RR//OER systems. The LSV curve of the CO_2_RR//HzOR system shows a significant decrease of the cell voltage to drive a current density of 10 mA cm^−2^ for CO formation and hydrazine decomposition, which is 0.71 V lower than the CO_2_RR//OER system, theoretically saving 38% energy. The durability test of H‐3DOM‐ZnN_4_/P‐C reveals that the FE_CO_ can be maintained at ≈85% during the 30 h electrolysis process (Figure 6h), confirming the excellent stability of H‐3DOM‐ZnN_4_/P‐C in CO_2_RR//HzOR system.

## 3 Conclusion

In summary, we have demonstrated pore engineering and microenvironment modulation of atomically dispersed ZnN_4_ sites for boosted CO_2_RR. The hollow‐wall and ordered macroporous structure can enhance the exposure of active sites, and the long‐range electronic regulation by P atoms endows the Zn center with high intrinsic activity and selectivity for CO_2_RR to CO. The as‐prepared H‐3DOM‐ZnN_4_/P‐C achieves ≈100% FE_CO_ at −0.6 V, and the FE_CO_ is higher than 90% at potentials range from −0.4 to −0.9 V. A ZCB based on H‐3DOM‐ZnN_4_/P‐C as cathode shows a peak power density of 5.3 mW cm^−2^, which could power red LED lights for practical applications. Moreover, we used the thermodynamically more favorable HzOR as an alternative reaction to replace OER to improve the overall CO_2_RR efficiency. Using H‐3DOM‐ZnN_4_/P‐C as anode and cathode materials, the CO_2_RR//HzOR electrolytic system saves 38% energy compared with the traditional CO_2_RR//OER system. This work opens up a new avenue to the optimization of single‐atom catalysts through pore engineering and electronic modulation for various energy‐related catalytic applications.

## Conflict of Interest

The authors declare no conflict of interest.

## Supporting information

Supporting Information

## Data Availability

The data that support the findings of this study are available from the corresponding author upon reasonable request.

## References

[1] a) J. Y. T. Kim , P. Zhu , F.‐Y. Chen , Z.‐Y. Wu , D. A. Cullen , H. Wang , Nat. Catal. 2022, 5, 288;

[2] a) S. Jin , Z. Hao , K. Zhang , Z. Yan , J. Chen , Angew. Chem., Int. Ed. 2021, 60, 20627; 10.1002/anie.202101818 33861487

[3] a) H. Zhang , W. Cheng , D. Luan , X. W. (.D.). Lou , Angew. Chem., Int. Ed. 2021, 60, 13177; 10.1002/anie.202014112 PMC8248387 33314631

[4] a) H. Fei , J. Dong , D. Chen , T. Hu , X. Duan , I. Shakir , Y. Huang , X. Duan , Chem. Soc. Rev. 2019, 48, 5207; 31573024 10.1039/c9cs00422j

[5] a) A. Dhakshinamoorthy , A. M. Asiri , H. Garcia , Adv. Mater. 2019, 31, 1900617; 10.1002/adma.201900617 31432586

[6] a) C. Hu , Y. Wang , J. Chen , H.‐F. Wang , K. Shen , K. Tang , L. Chen , Y. Li , Small 2022, 18, 2201391; 10.1002/smll.202201391 35523724

[7] a) T. Ding , X. Liu , Z. Tao , T. Liu , T. Chen , W. Zhang , X. Shen , D. Liu , S. Wang , B. Pang , D. Wu , L. Cao , L. Wang , T. Liu , Y. Li , H. Sheng , M. Zhu , T. Yao , J. Am. Chem. Soc. 2021, 143, 11317; 34293258 10.1021/jacs.1c05754

[8] a) J. Du , F. Li , L. Sun , Chem. Soc. Rev. 2021, 50, 2663; 33400745 10.1039/d0cs01191f

[9] a) G. Cai , P. Yan , L. Zhang , H.‐C. Zhou , H.‐L. Jiang , Chem. Rev. 2021, 121, 12278; 34280313 10.1021/acs.chemrev.1c00243

[10] a) Z. Zhu , H. Yin , Y. Wang , C.‐H. Chuang , L. Xing , M. Dong , Y.‐R. Lu , G. Casillas‐Garcia , Y. Zheng , S. Chen , Y. Dou , P. Liu , Q. Cheng , H. Zhao , Adv. Mater. 2020, 32, 2004670; 10.1002/adma.202004670 32939887

[11] a) K. Shen , L. Zhang , X. Chen , L. Liu , D. Zhang , Y. Han , J. Chen , J. Long , R. Luque , Y. Li , B. Chen , Science 2018, 359, 206; 29326271 10.1126/science.aao3403

[12] a) C. Hu , Y. Zhang , A. Hu , Y. Wang , X. Wei , K. Shen , L. Chen , Y. Li , Adv. Mater. 2023, 35, 2209298; 10.1002/adma.202209298 36843343

[13] a) W. Guo , X. Tan , J. Bi , L. Xu , D. Yang , C. Chen , Q. Zhu , J. Ma , A. Tayal , J. Ma , Y. Huang , X. Sun , S. Liu , B. Han , J. Am. Chem. Soc. 2021, 143, 6877; 33856799 10.1021/jacs.1c00151

[14] a) Y. Li , B. Wei , M. Zhu , J. Chen , Q. Jiang , B. Yang , Y. Hou , L. Lei , Z. Li , R. Zhang , Y. Lu , Adv. Mater. 2021, 33, 2102212; 10.1002/adma.202102212 34463377

[15] W. Xia , M. A. Hunter , J. Wang , G. Zhu , S. J. Warren , Y. Zhao , Y. Bando , D. J. Searles , Y. Yamauchi , J. Tang , Chem. Sci. 2020, 11, 9584. 34094224 10.1039/d0sc02518f PMC8162149

[16] X. Rong , H.‐J. Wang , X.‐L. Lu , R. Si , T.‐B. Lu , Angew. Chem., Int. Ed. 2020, 59, 1961. 10.1002/anie.201912458 31674119

[17] a) Y. Xin , K. Shen , T. Guo , L. Chen , Y. Li , Small 2023, 19, 2300019; 10.1002/smll.202300019 36840653

[18] M. Qiao , Y. Wang , Q. Wang , G. Hu , X. Mamat , S. Zhang , S. Wang , Angew. Chem., Int. Ed. 2020, 59, 2688. 10.1002/anie.201914123 31769154

[19] Q. Wang , T. Ina , W.‐T. Chen , L. Shang , F. Sun , S. Wei , D. Sun‐Waterhouse , S. G. Telfer , T. Zhang , G. I. N. Waterhouse , Sci. Bull. 2020, 65, 1743. 10.1016/j.scib.2020.06.020 36659247

[20] J. Feng , L. Wu , S. Liu , L. Xu , X. Song , L. Zhang , Q. Zhu , X. Kang , X. Sun , B. Han , J. Am. Chem. Soc. 2023, 145, 9857. 37092347 10.1021/jacs.3c02428

[21] J. Chen , T. Wang , X. Wang , B. Yang , X. Sang , S. Zheng , S. Yao , Z. Li , Q. Zhang , L. Lei , J. Xu , L. Dai , Y. Hou , Adv. Funct. Mater. 2022, 32, 2110174.

[22] T. Wang , Q. Wang , Y. Wang , Y. Da , W. Zhou , Y. Shao , D. Li , S. Zhan , J. Yuan , H. Wang , Angew. Chem., Int. Ed. 2019, 58, 13466. 10.1002/anie.201907752 31268612

