# Supplementary material for: Atomically Dispersed ZnN4 Sites Anchored on P‐Functionalized Carbon with Hierarchically Ordered Porous Structures for Boosted Electroreduction of CO2
Source: Adv Sci (Weinh). 2023 Dec 7;11(4):2306095. doi: 10.1002/advs.202306095 (PMC10811484; doi:10.1002/advs.202306095)
Supplement: Supplementary file 1 — Supporting Information [file ADVS-11-2306095-s001.pdf]

ADVANCED SCIENCE
Open Access

Supporting Information

for *Adv. Sci.*, DOI 10.1002/advs.202306095

Atomically Dispersed ZnN_4 Sites Anchored on P-Functionalized Carbon with Hierarchically Ordered Porous Structures for Boosted Electroreduction of CO_2

Chenghong Hu, Wen Yao, Xianfeng Yang, Kui Shen, Liyu Chen and Yingwei Li**

Supporting Information

Atomically dispersed ZnN_4 sites anchored on P-functionalized carbon with hierarchically ordered porous structures for boosted electroreduction of CO_2

Chenghong Hu, Wen Yao, Xianfeng Yang, Kui Shen, Liyu Chen,* Yingwei Li*

Experimental Section

Materials

$Zn(NO_3)_2 \cdot 6H_2O$, 2-methylimidazole, $KHCO_3$, and phytic acid solution were purchased from Aladdin Co., Ltd. CH_3OH was provided by Guangdong Guanghua Sci-Tech Co., Ltd. $K_2S_2O_8$, styrene, $NH_3 \cdot H_2O$ was supplied by Tianjin Damao chemical reagent Co., Ltd. All chemicals were directly used without further purification.

Catalyst preparation

Synthesis of three-dimensional-ordered polystyrene sphere (3D-ordered PS) template. Uniform PS spheres with a diameter of 410 nm were synthesized by soap-free polymerization. 52 mL of styrene was added to 400 mL of deionized H_2O followed by the addition of 0.43 g of potassium persulfate. The mixtures were heated at 85 °C for 6 h under nitrogen atmosphere. Monodispersed PS spheres with sizes of about 410 nm were obtained. A 40 mL of PS colloidal dispersion was centrifuged at a rate of 2 krpm for 24 h. The obtained precipitations were dried at 50 °C overnight to obtain the final 3D-ordered PS template.

Synthesis of 3DOM-ZIF-8. The 3D-ordered PS template was immersed into 10 mL of methanol solution containing 4.08 g of $Zn(NO_3)_2 \cdot 6H_2O$ and 3.38 g of 2-methylimidazole for 2 h, which was then degassed under vacuum for 30 min to assure the precursor solution to be entered into the voids of the template. The obtained 3D PS@precursor was transferred to a clean beaker and dried at 50 °C overnight. Next, the dry 3D PS@precursor was immersed in a mixed solution of $NH_3 \cdot H_2O/CH_3OH$ (1:1 V/V) and then degassed under vacuum for 30 min to ensure the mixed solution was homogeneously permeated. The above solution was left for complete crystallization for 24 h. ZIF-8@PS was obtained after washing with methanol several times and drying at

50 °C for 6 h. Finally, the 3DOM-ZIF-8 material was successfully prepared through simple calcination of the ZIF-8@PS at 400 °C for 5 h.

Synthesis of ZIF-8. Typically, 2.9 g $Zn(NO_3)_2\cdot 6H_2O$ was dissolved in 150 mL methanol to prepare solution A; 9.6 g 2-methylimidazole and was dissolved in 150 mL methanol to prepare solution B. Subsequently, solution A was added to solution B, and kept stirring for 12 h at room temperate. After centrifugation, the final product was dried at 60 °C for 5 h.

Preparation of H-3DOM-ZnN_4/P-C. 100 mg 3DOM-ZIF-8 was added into 50 mL methanol solution containing 100 mg phytic acid solution, and then stirred in an oil bath at 45 °C for 2 h. After filtration, the solid was placed in an oven and dried at 60 °C for 8 h to afford H-3DOM-ZIF-8/Zn-PA. Then, the H-3DOM-ZIF-8/Zn-PA was heated at 950 °C for 2 h in a tubular furnace with a heating rate of 5 °C min^{-1} under flowing Ar atmosphere. After cooling to room temperature, the final product H-3DOM-ZnN_4/P-C was obtained.

Preparation of 3DOM-ZnN_4/C. 100 mg 3DOM-ZIF-8 was heated at 950 °C for 2 h in a tubular furnace with a heating rate of 5 °C min^{-1} under flowing Ar atmosphere. After cooling to room temperature, the product 3DOM-ZnN_4/C was obtained.

Preparation of 3DOM-ZnN_4/P-C. 100 mg 3DOM-ZnN_4/C was added into 50 mL methanol solution containing 100 mg phytic acid solution, which was stirred in an oil bath at 45 °C for 2 h. After filtration, the black solid was placed in an oven at 60 °C and dried for 8 h. Then, the black solid was heated at 950 °C for 30 min in a tubular furnace with a heating rate of 5 °C min^{-1} under flowing Ar atmosphere. After cooling to room temperature, the product 3DOM-ZnN_4/P-C was obtained.

Preparation of ZnN_4/P-C. The preparation process is the same as that of 3DOM-ZnN_4/P-C, except that 3DOM-ZIF-8 was replaced by conventional ZIF-8.

Catalyst characterization

Powder X-ray diffraction (XRD) patterns of the samples were collected with a Rigaku diffractometer (D/MAX/IIIA, 3 kW) employing Cu Kα radiation (40 kV, 30 mA, λ = 0.1543 nm). Raman spectra were recorded on a LabRAM Aramis Raman spectrometer (HORIBA Jobin Yvon). N_2 adsorption/desorption isotherms were obtained at 77 K on an ASAP 2460 instrument. The morphology of the materials was investigated by high-

resolution scanning electron microscopy (SEM, SU 8220, and 8100 of HITACHI). The structure and element mapping was determined by a high-resolution transmission electron microscope (TEM, JEOL, JEM-2100F) with EDS analysis (Bruker Xflash 5030T) operated at 200 kV. The atomic structure of the catalyst was characterized using a Titan Cubed Themis G2300 (FEI, Netherlands) transmission electron microscope operated equipped with double spherical aberration correctors at 200 kV. X-ray photoelectron spectroscopy (XPS) was performed by using a Thermo Scientific Escalab 250Xi system with a base pressure of 2×10^{-9} Torr. Synchrotron-based X-ray absorption fine structure (XAFS) spectra at the Zn K-edge were collected at BL14W1 station in Shanghai Synchrotron Radiation Facility (SSRF). The metal contents of the samples were determined by atomic absorption spectroscopy (AAS) on a HITACHI Z-2300 instrument. The C, N, and P elemental contents of the samples were measured on a Euro Vector EA3000 instrument and Inductively coupled plasma-optical emission spectrometer (ICP-OES, Optima 8300, PE). Static contact angles (CA) were measured on a Dataphysics OCA40 Micro instrument. The Fourier transform infrared spectroscopy (FT-IR) of the samples was measured on a Nicolet IS10. The Zeta potential was determined by Zetasizer ULTRA.

Electrochemical measurements

Electrode preparation. Typically, 1 mg of catalyst was suspended in 250 μL of CH_3CH_2OH and dispersed uniformly by ultrasonic treatment. Then, the solution was spread onto the carbon cloth (1×1 cm^2) surface by a micropipette. Finally, 10 μL of Nafion D-521 dispersion (0.5 wt%) was pipetted onto the carbon cloth to immobilize the catalyst.

Electrocatalytic CO_2 reduction. All the electrochemical experiments were conducted on the electrochemical workstation (CHI 760E). All potentials cited in this work were referenced to the RHE, unless stated otherwise. The reference potentials were converted to RHE using the formulas $E_{(vs.\ RHE)} = E_{(vs.\ Ag/AgCl)} + 0.197\ V + 0.059 \times pH$. The electrolysis experiments were conducted at 25 °C in an H-type cell with a working cathode, a counter anode (platinum gauze), and a reference electrode (Ag/AgCl with saturated KCl). In the experiment, Nafion-117 membrane was used as the proton

exchange membrane that separated the cathode and anode compartments. $KHCO_3$ aqueous solution (0.1 M) was utilized as the electrolyte. In each experiment, 35 mL of the electrolyte was used. Before starting the electrolysis experiment, the catholyte was bubbled with CO_2 for 30 min to ensure the solution is saturated.

Product analysis. The gaseous product of electrochemical experiments was analyzed by gas chromatography (GC, HP 4890D), which was equipped with FID and TCD detectors using helium as the internal standard. The liquid product was analyzed by ^1H NMR (Bruker Avance III 400 HD spectrometer) in deuterium oxide.

The turnover frequency (TOF) value of CO_2RR was calculated according to the equation:

$$TOF = j_{CO} / (n \times N \times F)$$

j_{CO} represents the current density of the CO (mA cm^{-2}). n is electron transfer number, which is 2 for CO. F is the Faraday constant (96485 C mol^{-1}), and N (mol) is the number of active sites involved in the CO_2RR. N can be calculated by the equation:

$$N = M/65.4 \times (ECSA / BET)$$

65.4 represents the relative atomic mass of Zn.

M is the mass of Zn in the catalyst on the electrode surface:

$$M = m_{cat} \times w$$

m_{cat} is mass of the catalyst on the electrode (mg), w (%) is the content of single-atom metal in the catalyst.

ECSA can be calculated by the equation:

$$ECSA = (C_{dl} / C_s) \times A_{geo}$$

C_{dl} is the double-layer capacitance value. C_s is the specific capacitance (typically 0.04 mF cm^{-2}) and A_{geo} is the geometric surface area of the electrode (1 cm^2)

Electrocatalytic hydrazine hydrate oxidation. The test device was consistent with CO_2 reduction, except that the electrolyte is replaced with 1 M KOH. Unless otherwise specified, the concentration of hydrazine hydrate is 100 mM.

The Energy saving efficiency (*η*) is calculated from the perspective of the energy of the full cell. The η value is calculated from the equation:

$$\eta = \frac{E_{CO2RR//OER} - E_{CO2RR//HzOR}}{E_{CO2RR//OER}} \times 100\%$$

Computational method

The free energies of CO_2 reduction states were carried out by the Vienna Ab-initio Simulation Package (VASP)[1,2], taking advantage of the density functional theory (DFT) with the Projected Augmented Wave (PAW) method[3]. The revised Perdew-Burke-Ernzerh of (RPBE) functional was used to describe the exchange and correlation effects [4-6]. For all the geometry optimizations, the cut-off energy was set to 450 eV. The Monkhorst-Pack grids[7] were set to be 3×3×1 and 5×5×1 for adsorption calculations and density of states (DOS) calculations, respectively. A 20 Å vacuum thickness was added in the z-direction of the simulation box, preventing interactions between the adjacent slabs.

In aqueous conditions, the reduction of CO_2 to produce CO could occur in the following three elementary steps:

$$CO_2 + (H^+ + e^-) + * \rightarrow *COOH$$

$$*COOH + (H^+ + e^-) \rightarrow *CO + H_2O$$

$$*CO \rightarrow CO + *$$

where * denotes the active sites on the catalyst surface. Based on the above mechanism, the free energy of two intermediate states, *COOH and *CO, are important to identify a given material's activity in catalyzing CO_2 reduction. The computational hydrogen electrode (CHE) model[8] proposed by Norskov et al. was used to calculate the free energies of CO_2 reduction intermediates, based on which the free energy of an adsorbed species is defined as

$$\Delta G_{ads} = \Delta E_{ads} + \Delta E_{ZPE} - T\Delta S_{ads} + \int C_P dT$$

where ΔE_{ads} is the electronic adsorption energy, ΔE_{ZPE} is the zero-point energy difference between adsorbed and gaseous species, $T\Delta S_{ads}$ is the corresponding entropy difference between these two states, and $\int C_P dT$ is the enthalpy correction. The electronic binding energy is referenced as graphene for each C atom, ½ H_2 for each H

atom, and (H_2O-H_2) for each O atom, plus the energy of the clean slab. The corrections of zero-point energy, entropy, and enthalpy of adsorbed and gaseous species can be found in the supporting information.

Supporting Figures and Tables

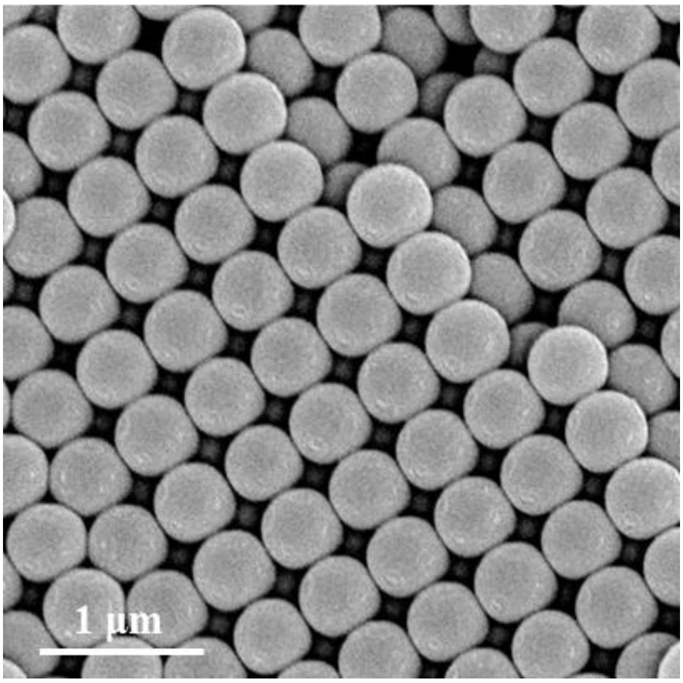

Figure S1. SEM image of PS template.

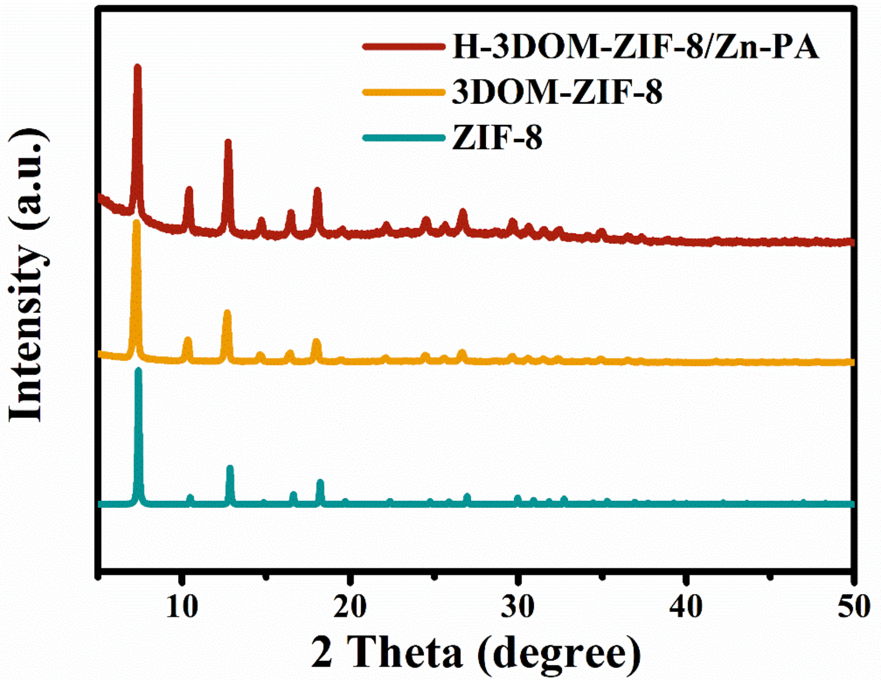

Figure S2. XRD patterns ZIF-8, 3DOM-ZIF-8, and H-3DOM-ZIF-8/Zn-PA.

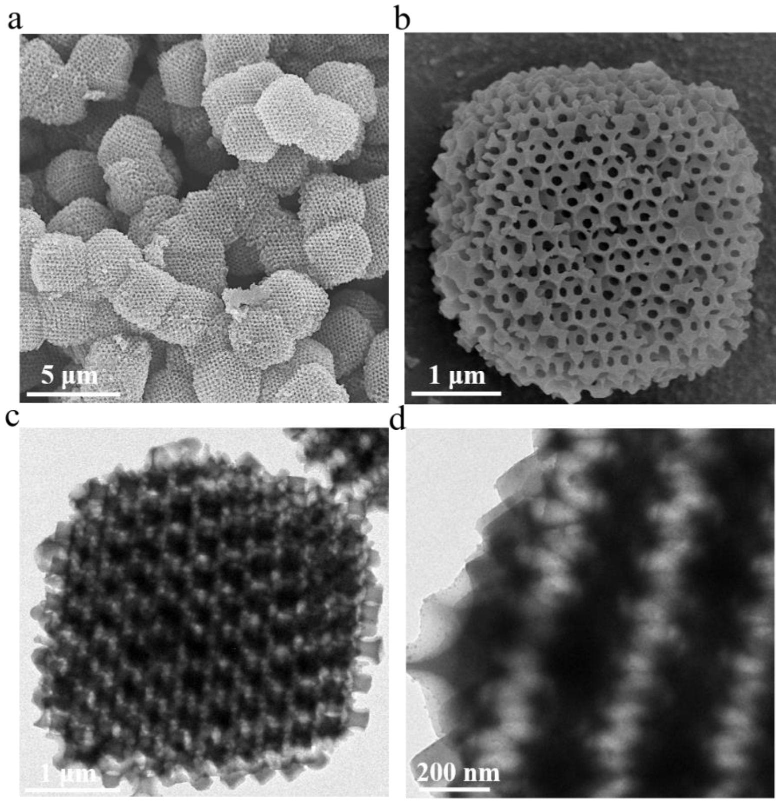

Figure S3. (a, b) SEM and (c, d) TEM images of 3DOM-ZIF-8 with different magnifications.

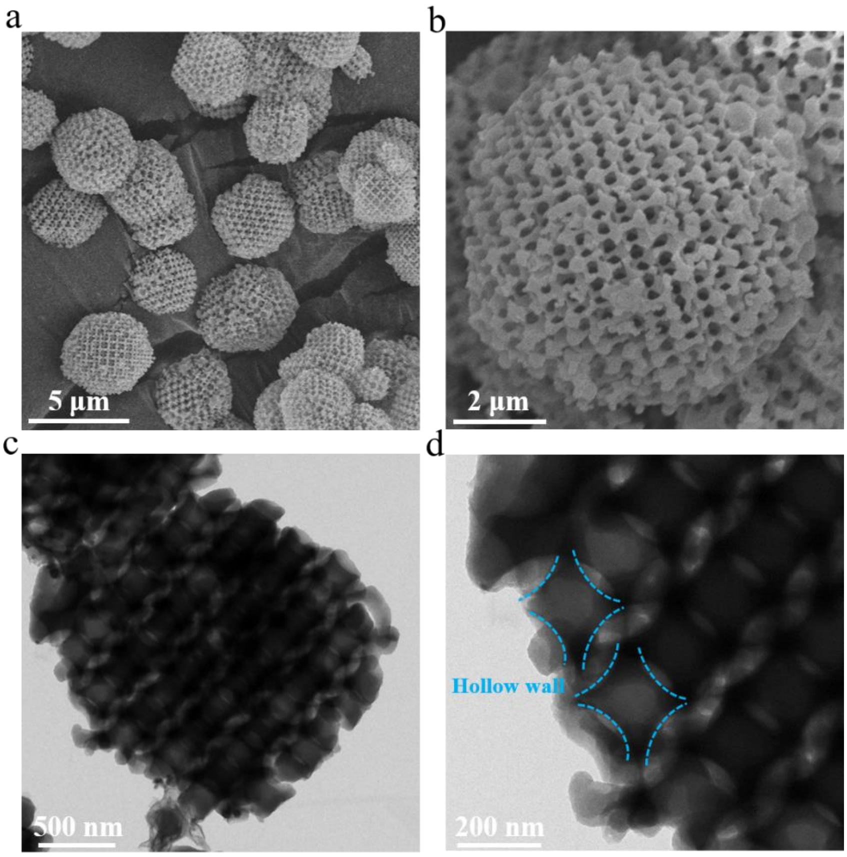

Figure S4. (a, b) SEM and (c, d) TEM images of H-3DOM-ZIF-8/Zn-PA with different magnifications.

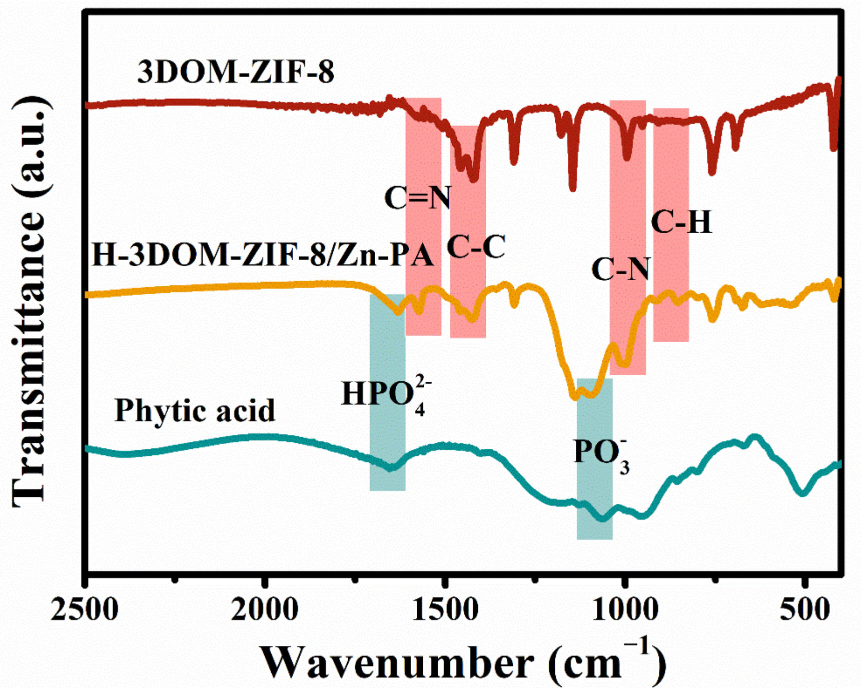

Figure S5. FTIR spectra of 3DOM-ZIF-8, H-3DOM-ZIF-8/Zn-PA, and phytic acid.

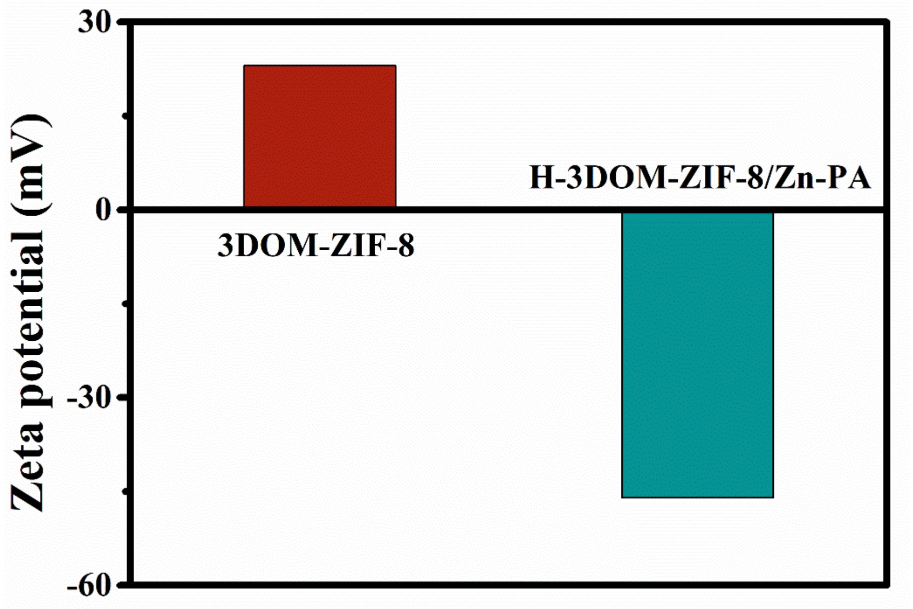

Figure S6. The zeta-potentials of 3DOM-ZIF-8 and H-3DOM-ZIF-8/Zn-PA.

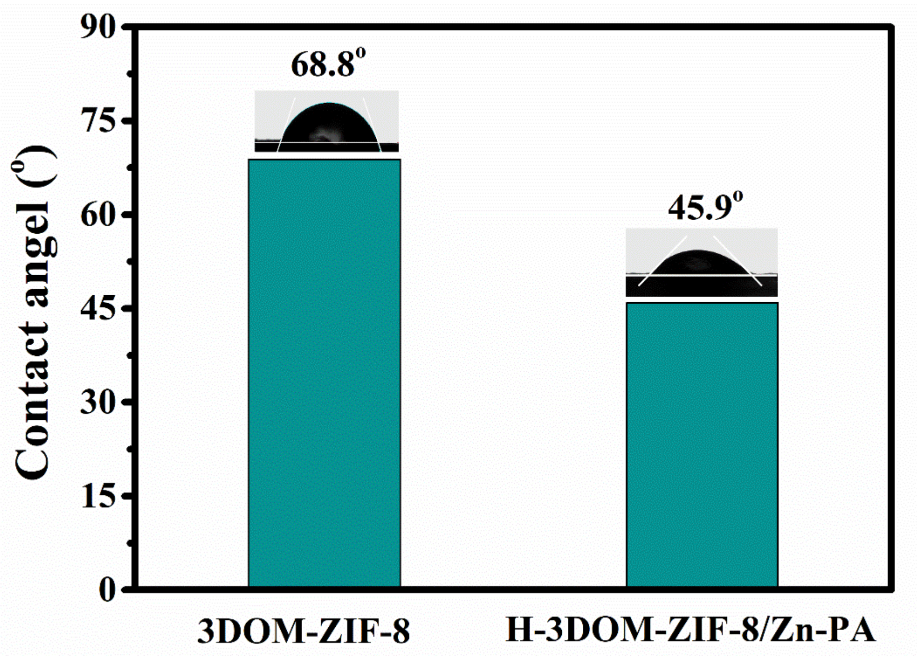

Figure S7. Water contact angle of the 3DOM-ZIF-8 and H-3DOM-ZIF-8/Zn-PA.

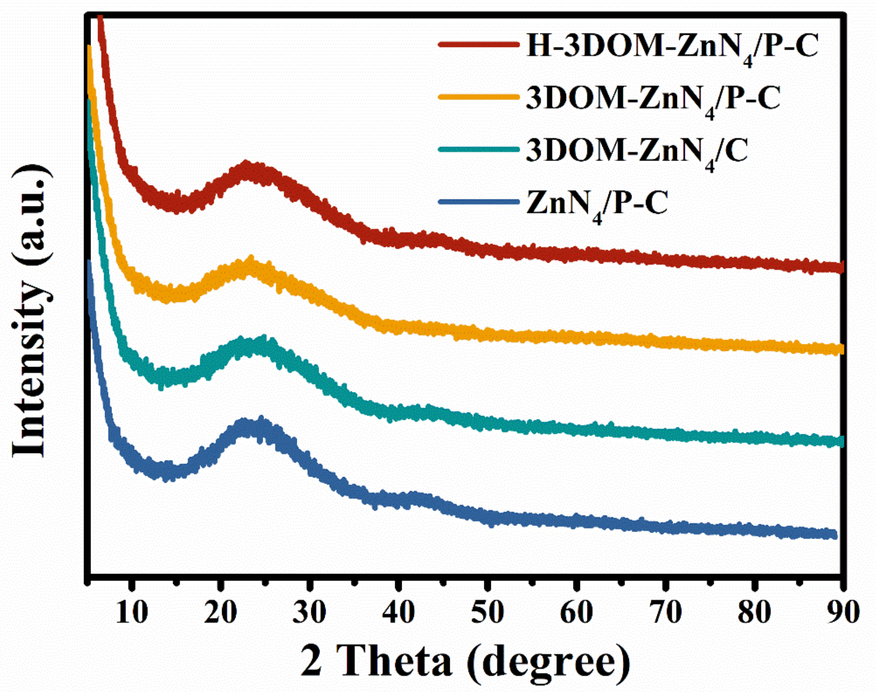

Figure S8. XRD patterns of different catalysts.

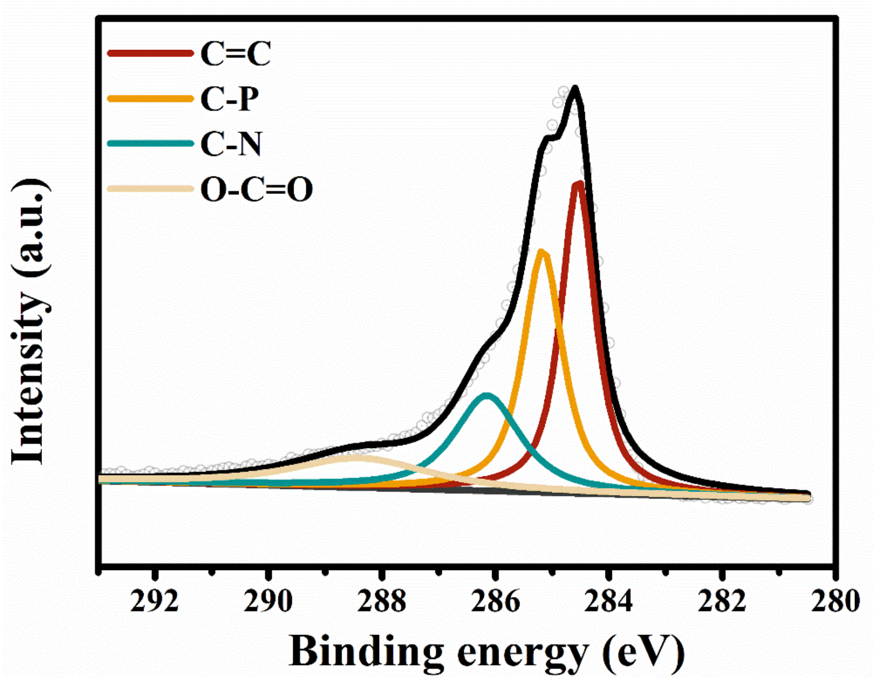

Figure S9. C 1s XPS spectra of H-3DOM-ZnN_4/P-C.

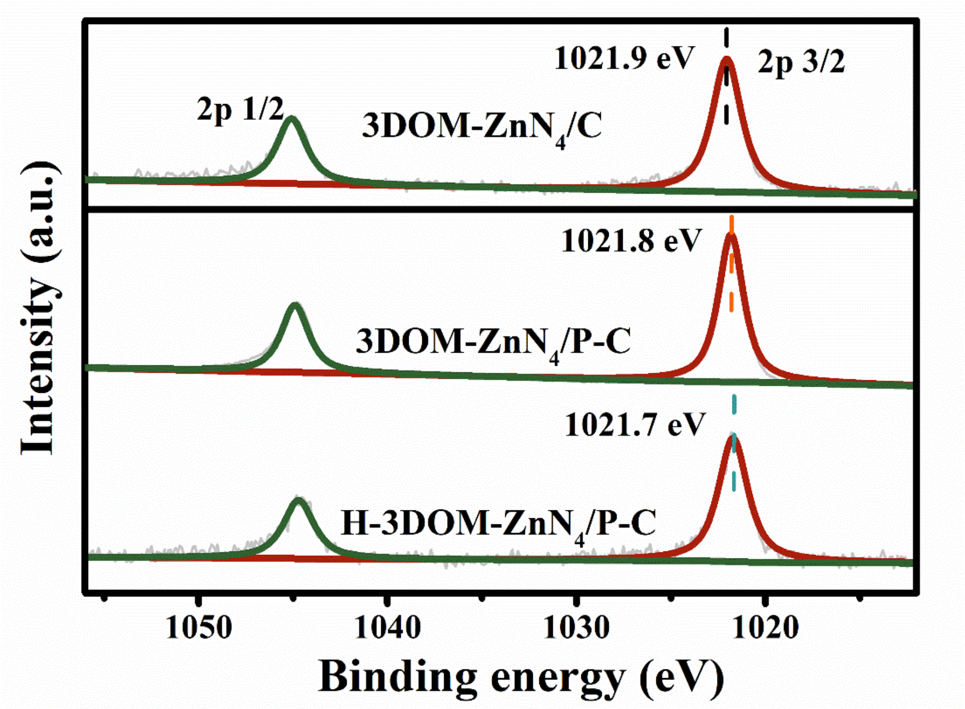

Figure S10. Zn 2p XPS spectra of H-3DOM-ZnN_4/P-C, 3DOM-ZnN_4/P-C, and 3DOM-ZnN_4/C.

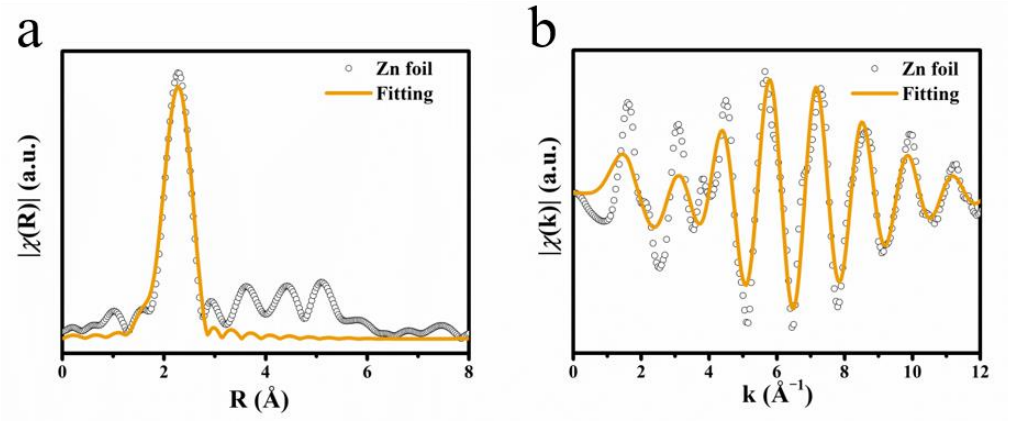

Figure S11. EXAFS fitting analysis of Zn foil in R (a) and k (b) space.

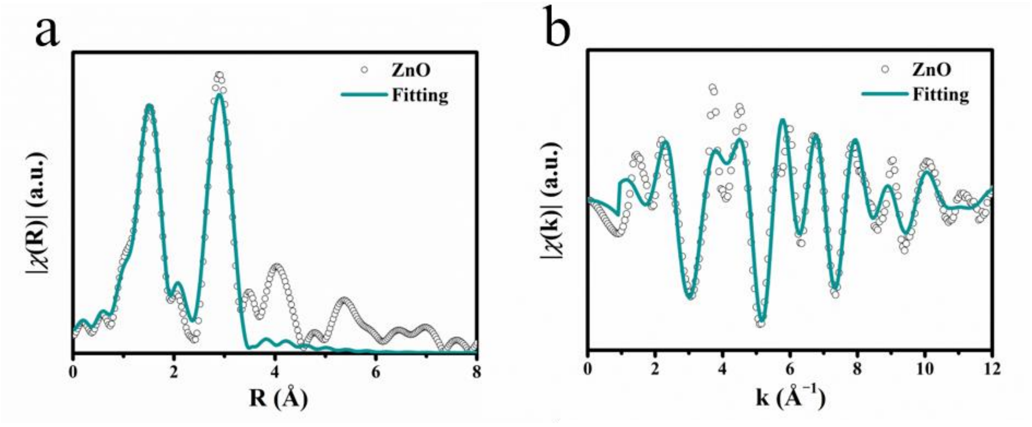

Figure S12. EXAFS fitting analysis of ZnO in R (a) and k (b) space.

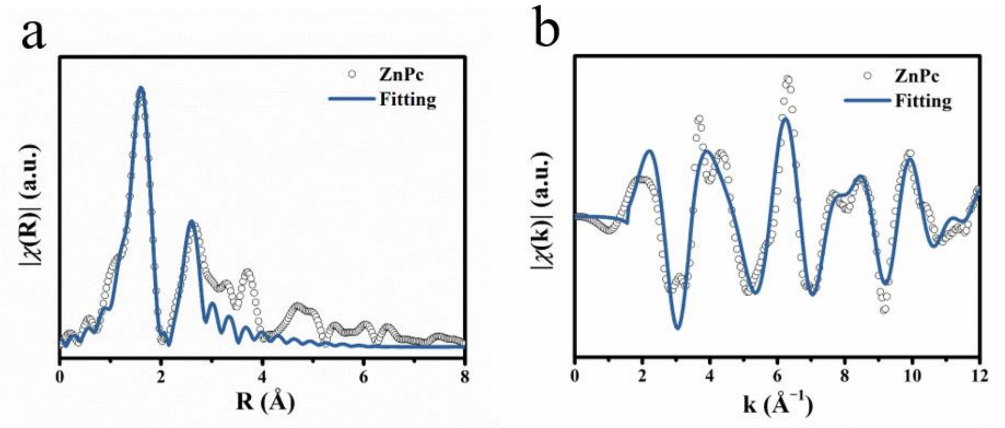

Figure S13. EXAFS fitting analysis of ZnPc in R (a) and k (b) space.

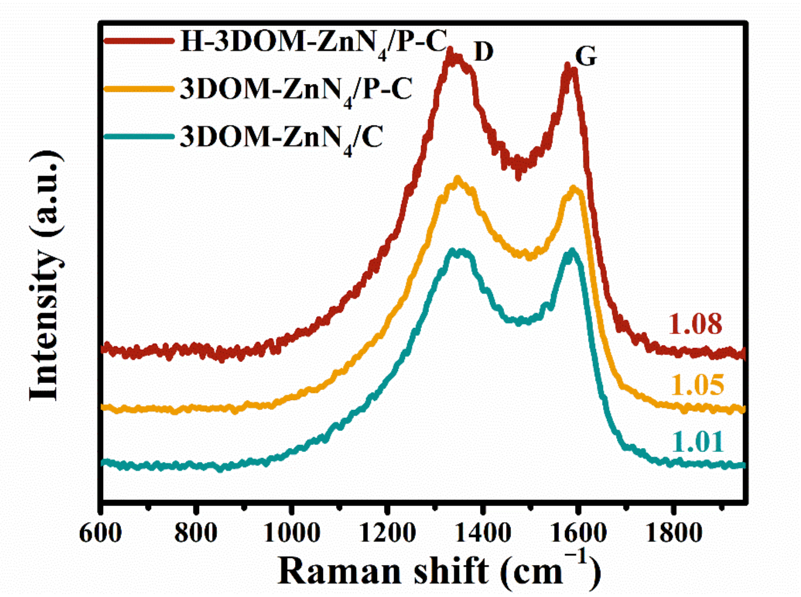

Figure S14. Raman spectra of 3DOM-ZnN_4/C, 3DOM-ZnN_4/P-C, and H-3DOM-ZnN_4/P-C.

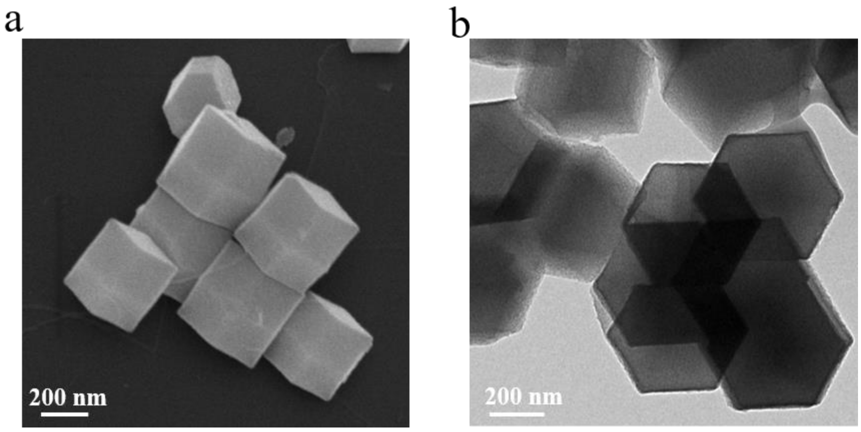

Figure S15. (a) SEM and (b) TEM images of ZnN_4/P-C.

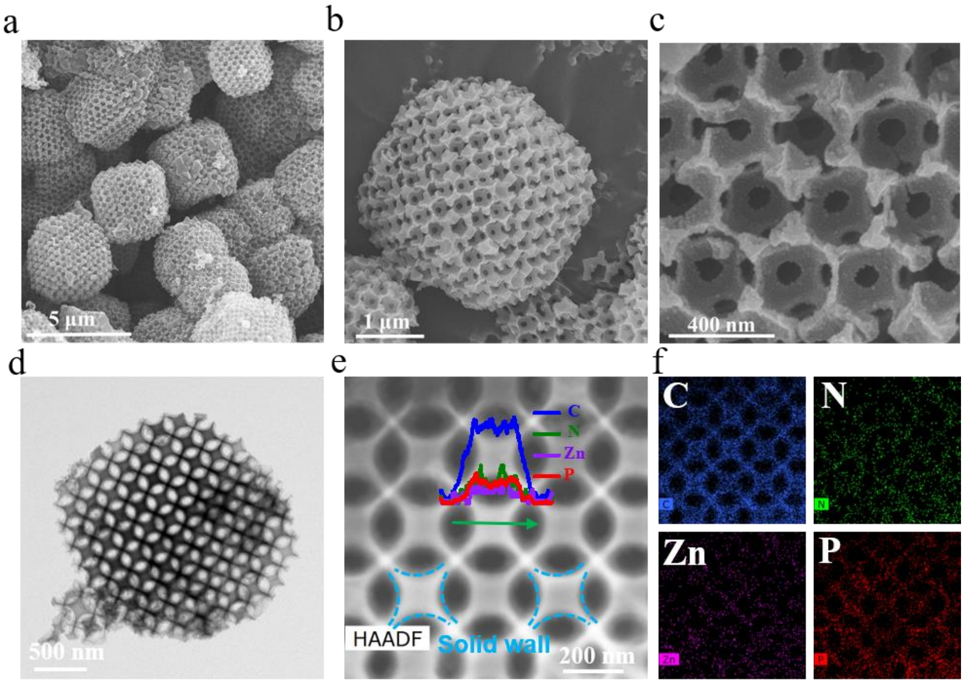

Figure S16. (a–c) SEM images of 3DOM-ZnN_4/P-C with different magnifications. (d) TEM image of 3DOM-ZnN_4/P-C. (e) HAADF-STEM image, elemental line scan profile (inset in e), and (f) corresponding EDS element mappings of 3DOM-ZnN_4/P-C.

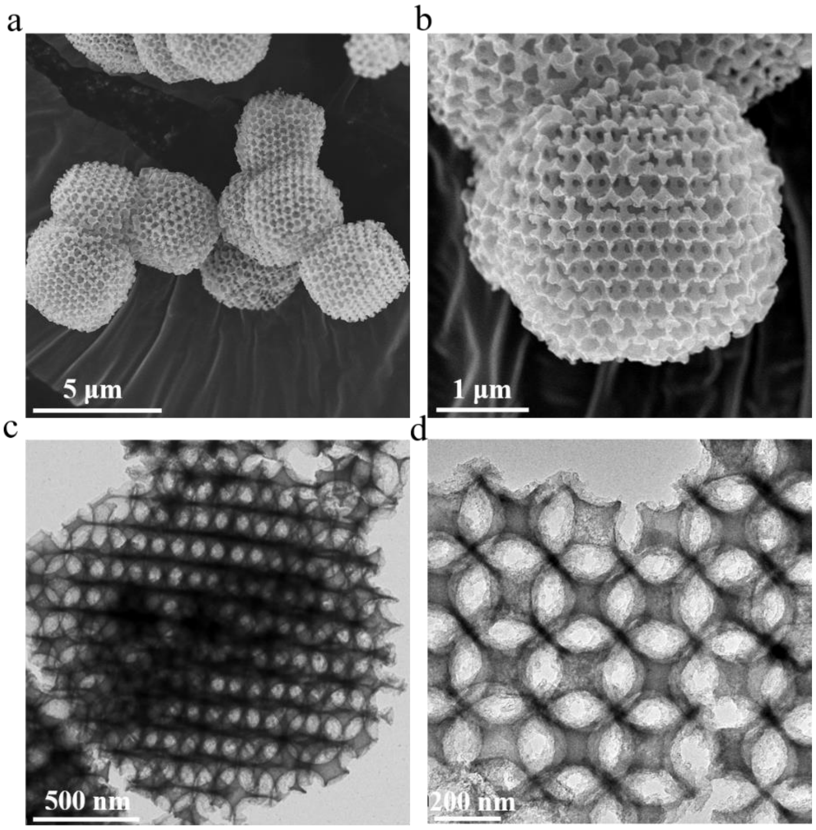

Figure S17. (a, b) SEM and (c, d) TEM images of 3DOM-ZnN_4/C with different magnifications.

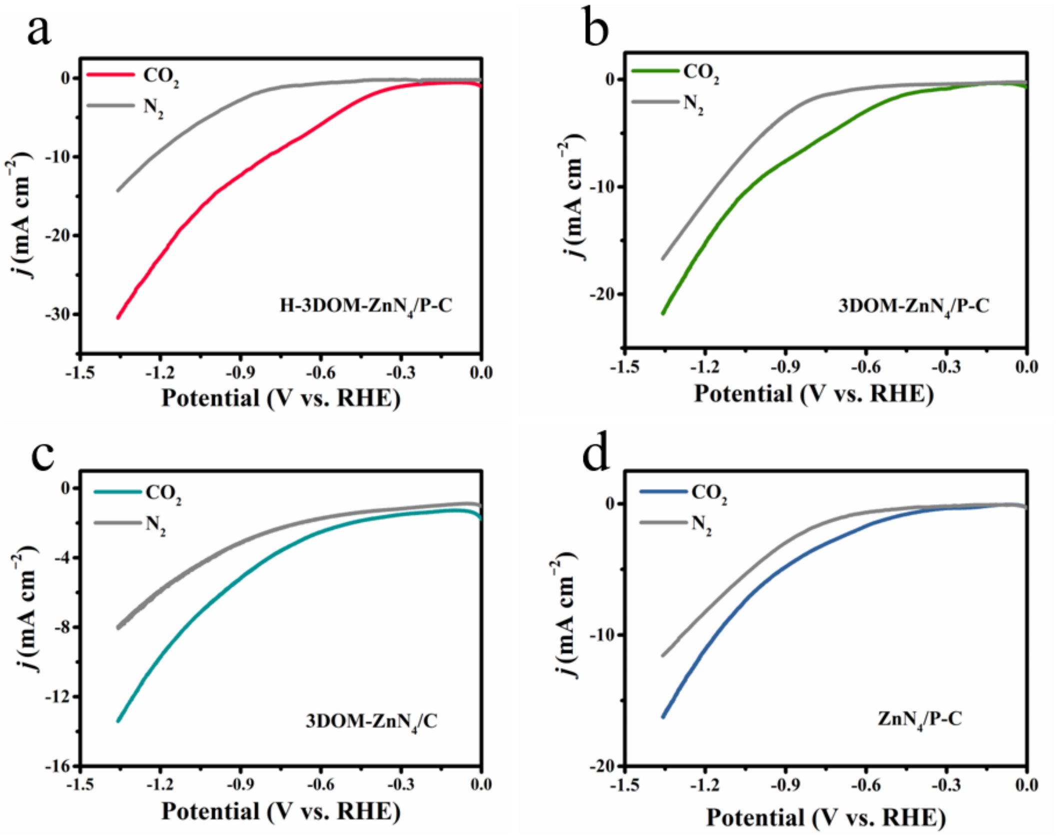

Figure S18. LSV curves of different catalysts in CO_2 and N_2-saturated 0.1 M $KHCO_3$.

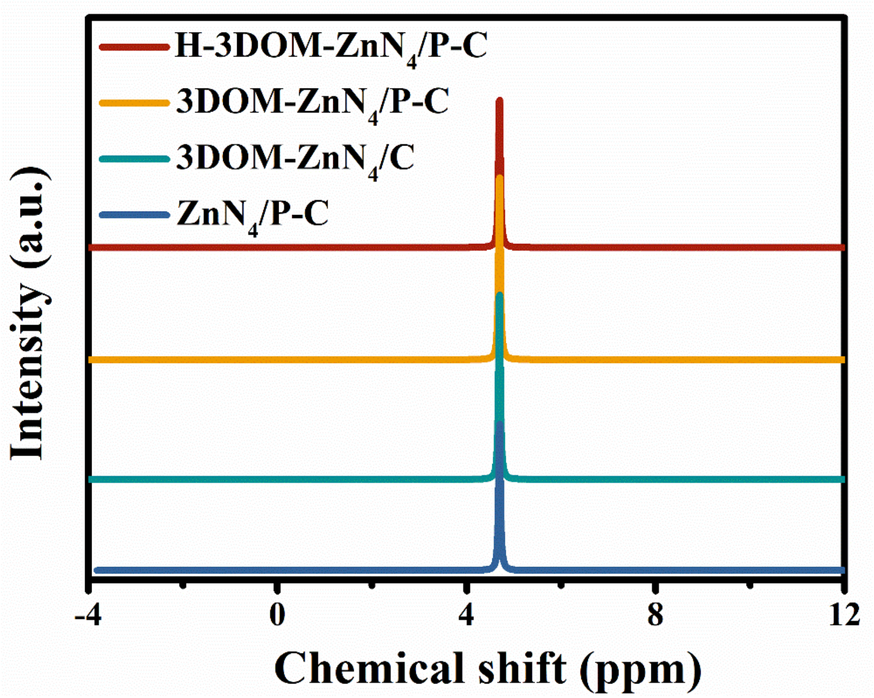

Figure S19. ^1H NMR spectra of the electrolyte after electrolysis at −0.6 V vs. RHE.

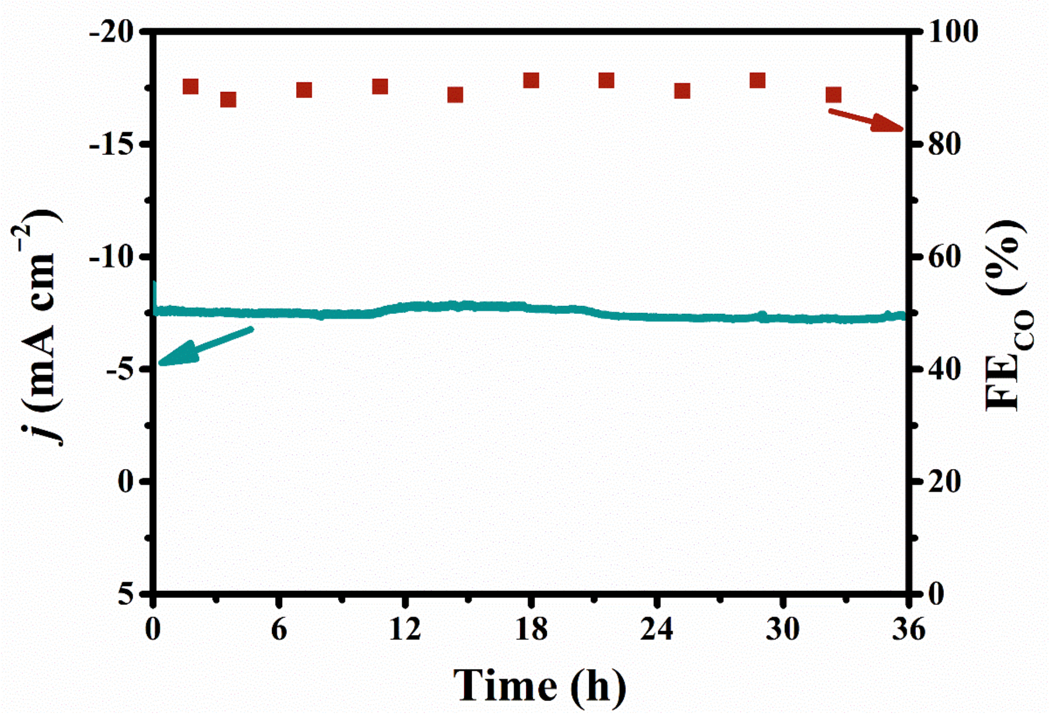

Figure S20. Amperometric i−t curve and FE_{CO} of H-3DOM-ZnN_4/P-C at −0.9 V vs. RHE.

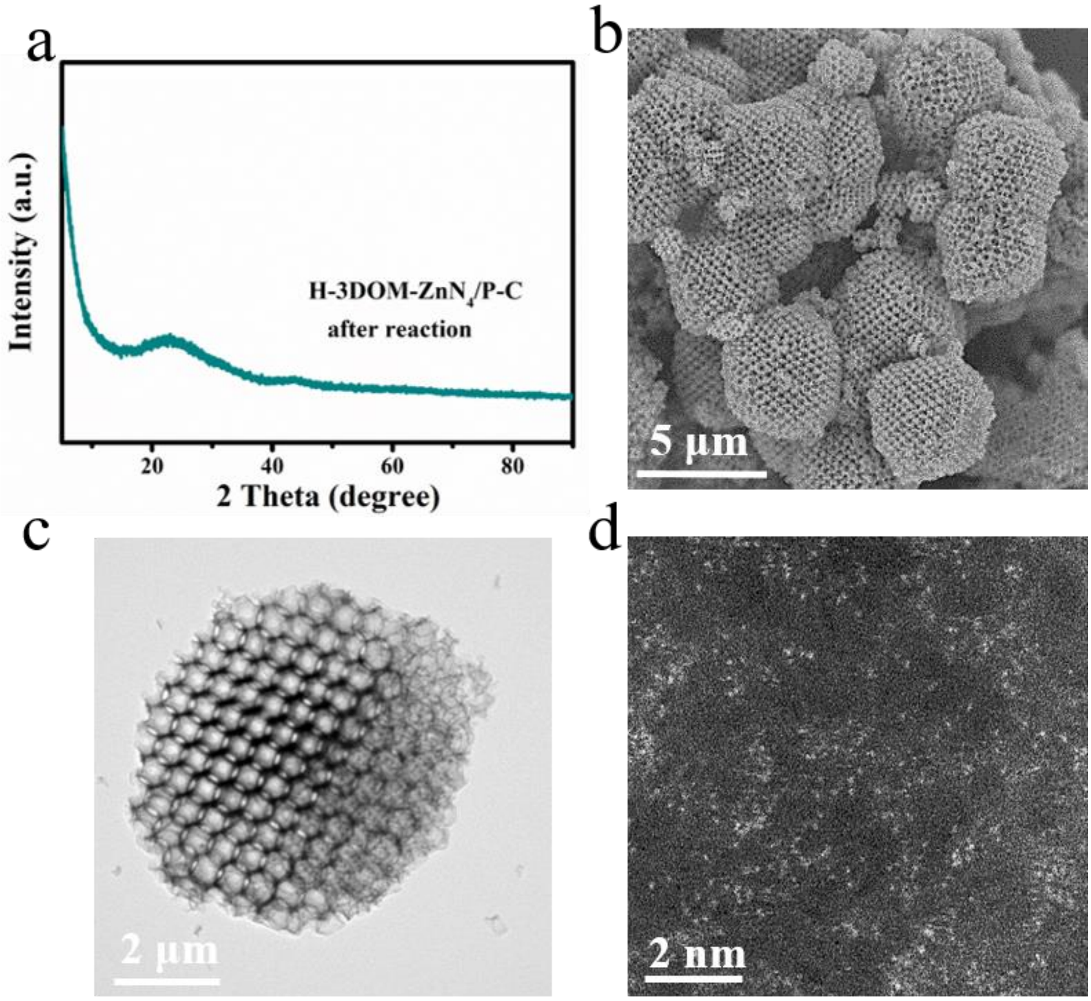

Figure S21. (a) XRD patterns, (b) SEM, (c) TEM, and (d) aberration-corrected HAADF-STEM images of H-3DOM-ZnN_4/P-C after the electrochemical CO_2RR.

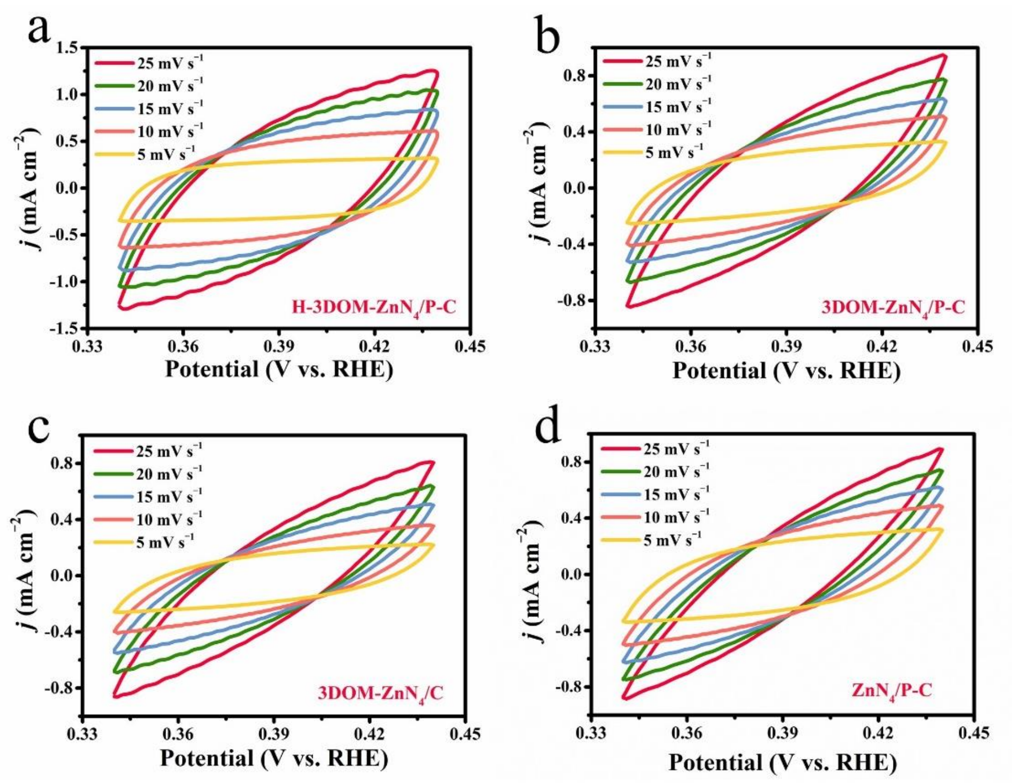

Figure S22. Measured CVs of (a) H-3DOM-ZnN_4/P-C, (b) 3DOM-ZnN_4/P-C, (c) 3DOM-ZnN_4/C, and (d) ZnN_4/P-C in 0.1 M $KHCO_3$ electrolyte at different scan rates.

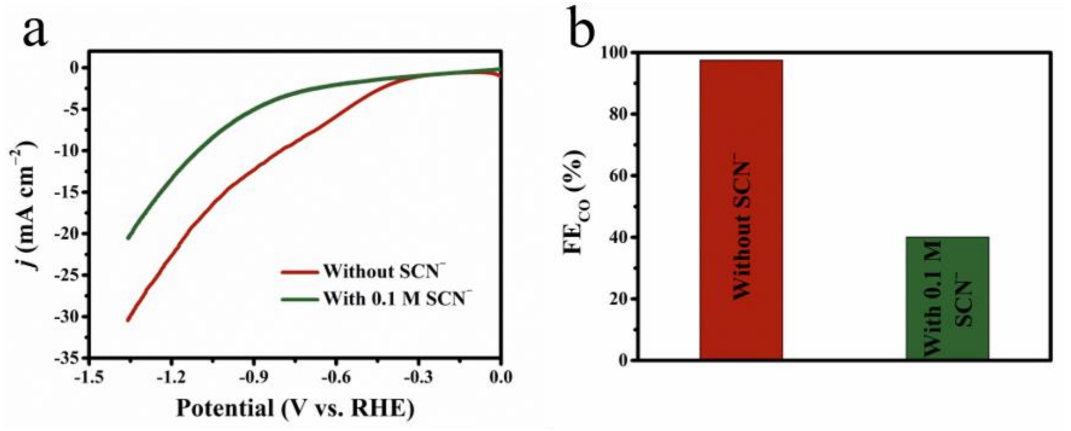

Figure S23. The LSV curve (a) and FE_{CO} (b) of H-3DOM-ZnN_4/P-C before and after adding SCN^-.

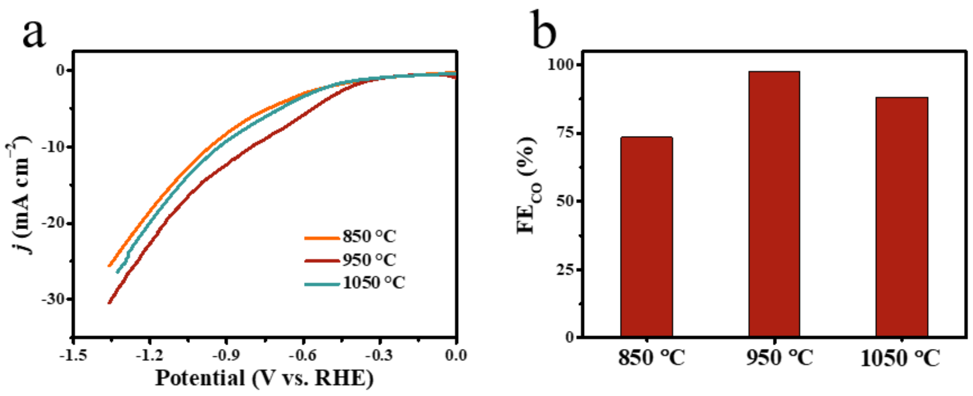

Figure S24. (a) LSV curves and (b) FE_{CO} at −0.6 V (vs. RHE) of different catalysts obtained at different calcination temperatures.

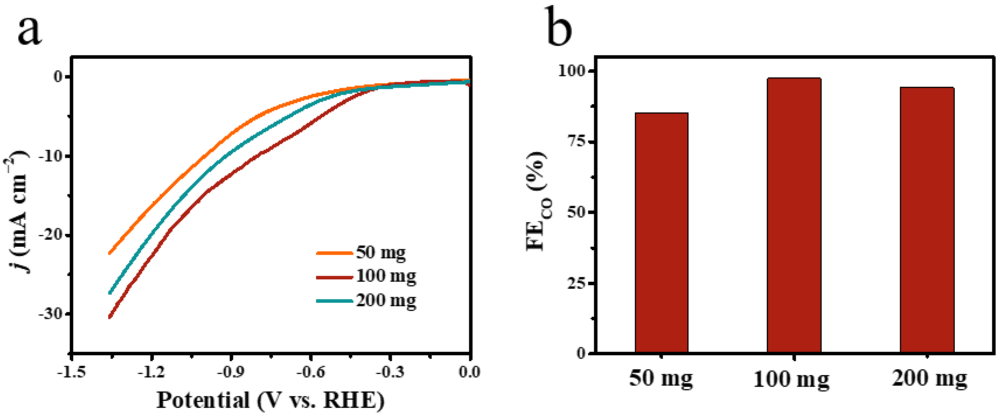

Figure S25. (a) LSV curves and (b) FE_{CO} at −0.6 V (vs. RHE) of different catalysts obtained with different amounts of phytic acid.

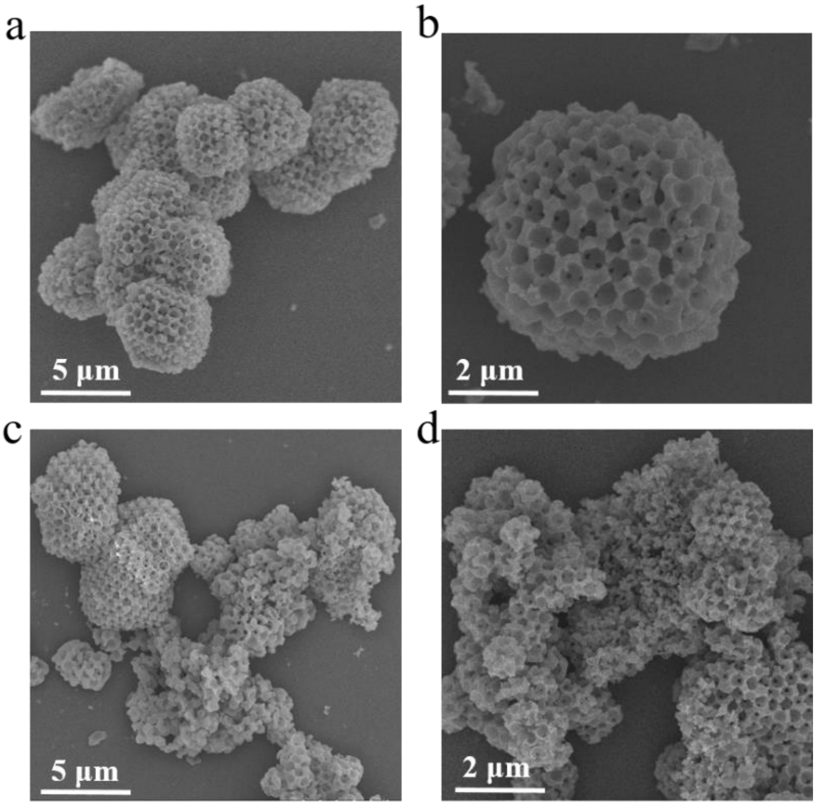

Figure S26. SEM images of 3DOM-ZIF-8/Zn-PA obtained with different amounts of phytic acid. (a, b) 50 mg and (c, d) 200 mg.

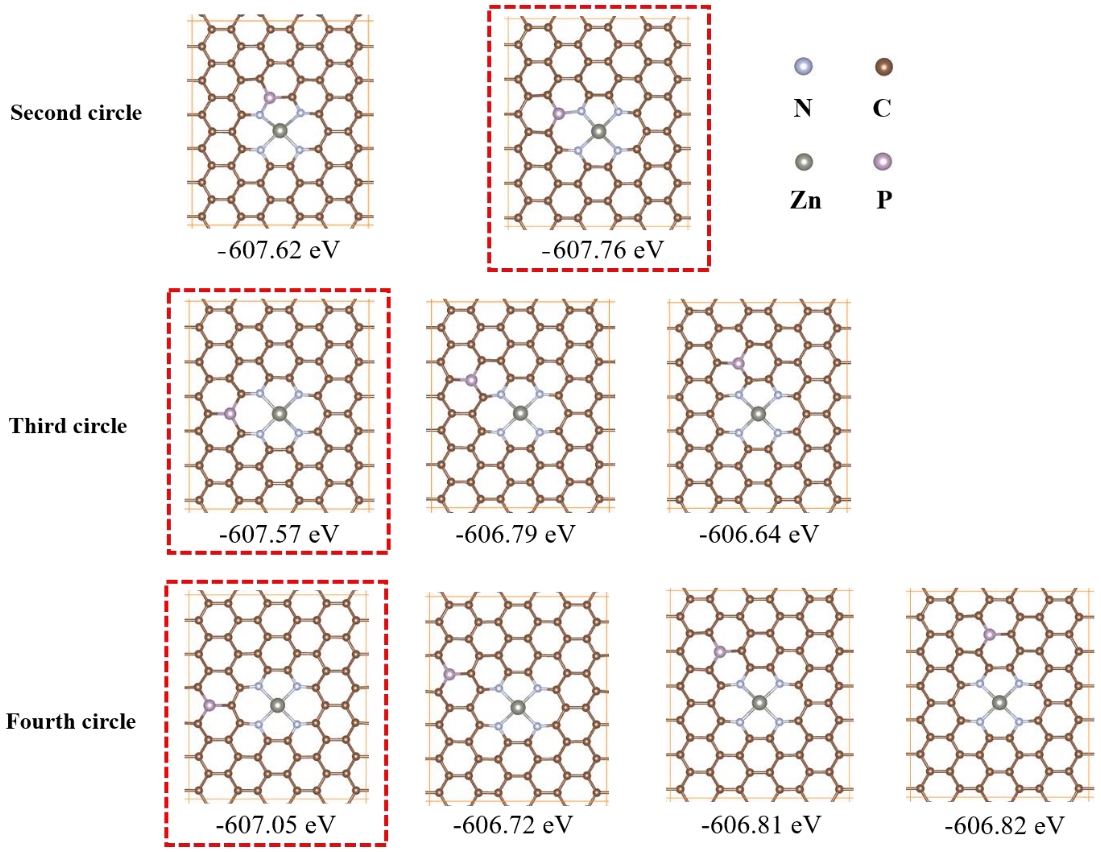

Figure S27. Local structures of ZnN_4/P graphene with phosphorous atom in various possible locations evaluated by DFT calculation.

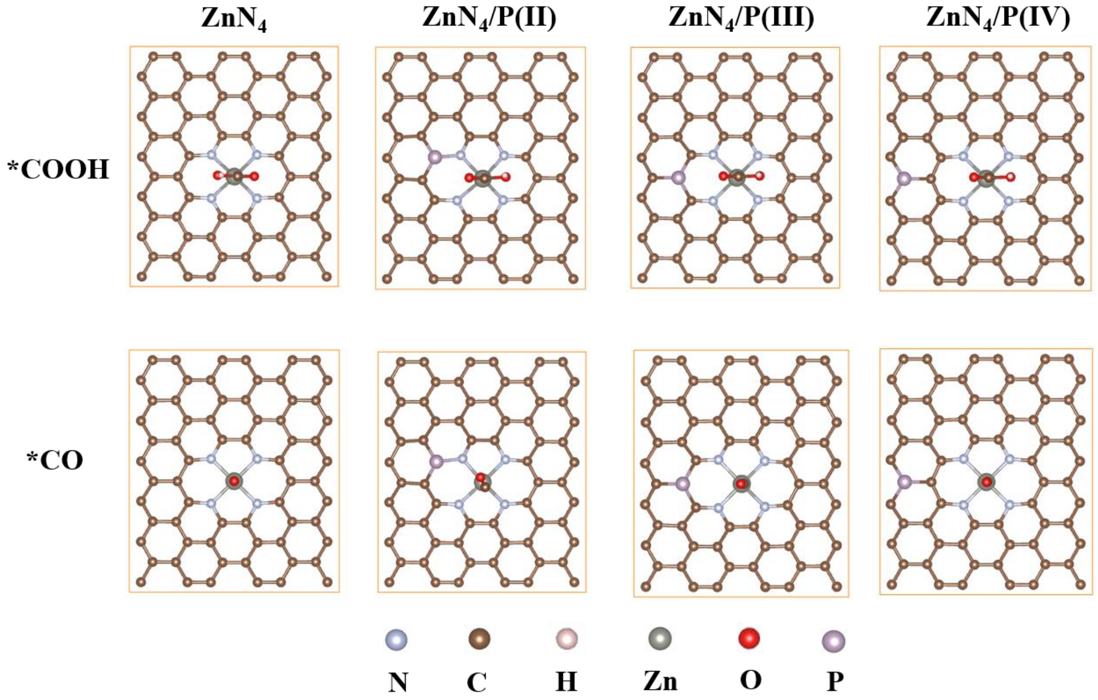

Figure S28. Model diagram of catalyst binding with intermediate states in CO_2RR process.

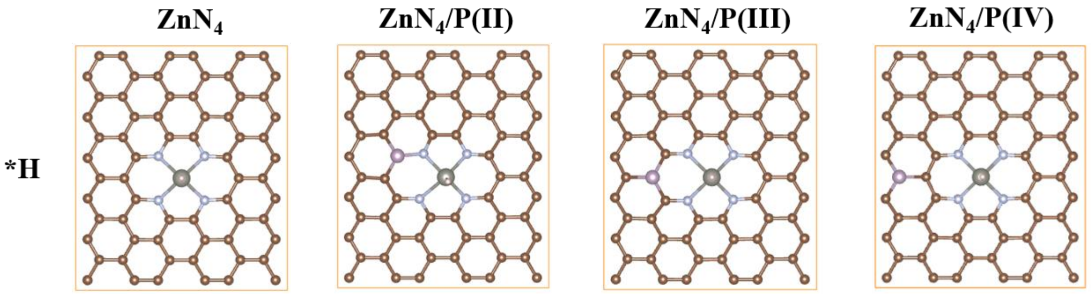

Figure S29. Model diagram of catalyst binding with intermediate states in HER process.

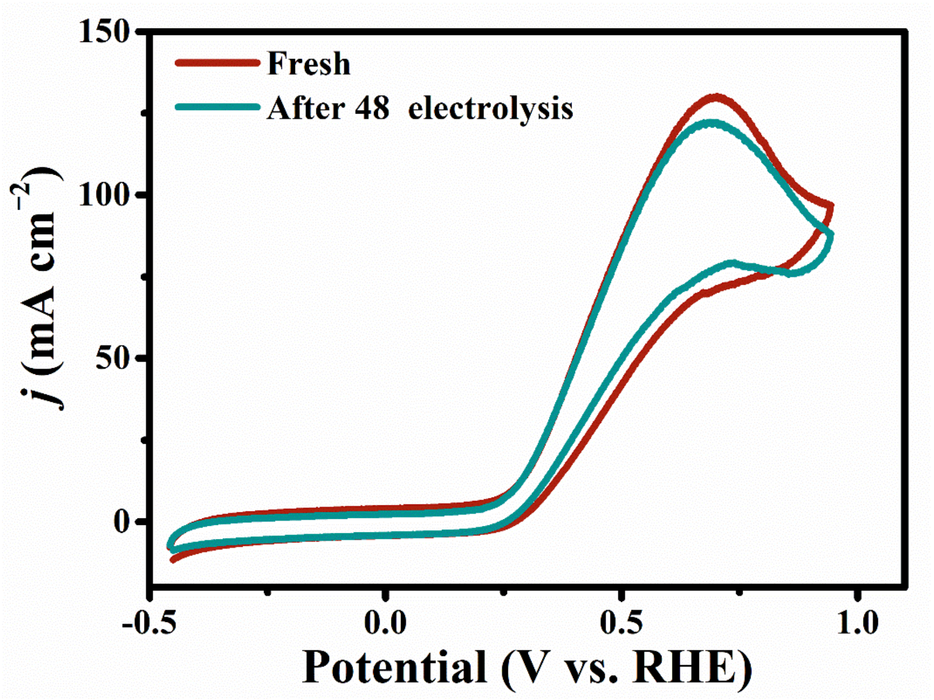

Figure S30. CV comparison before and after long-term electrolysis.

Table S1. The pH value of methanol and PA-methanol solution.

Solution	pH	Concentration of H^+ ($mol \cdot L^{-1}$)
Methanol	7.8	/
PA-methanol	1.6	0.025

Table S2. The Zn, N, and P loadings of different catalysts measured by AAS and ICP-OES.

Sample	Zn contents (wt%)	P contents (wt%)	N contents (wt%)
**H-3DOM-ZnN_4/P-C**	1.56	0.98	6.73
**3DOM-ZnN_4/P-C**	1.88	1.86	7.15
**3DOM-ZnN_4/C**	2.03	/	8.28
**ZnN_4/P-C**	1.98	1.21	6.87

Table S3. Structural parameters of EXAFS fitting of H-3DOM-ZnN_4/P-C.

Catalyst	Path	CN	R (Å)	$\sigma^2(10^{-3}\ Å^2)$	ΔE_0 (eV)	R-factor
**H-3DOM-ZnN_4/P-C**	Zn-N	4.0±0.7	2.04±0.02	2.9±2.2	3.9±1.6	0.02

CN: coordination numbers; *R*: bond distance; σ^2: Debye-Waller factors; ΔE_0: the inner potential correction. *R* factor: goodness of fit.

Table S4. Comparison of CO_2RR performances of H-3DOM-ZnN_4/P-C with other reported single-atom catalysts.

Catalysts	Electrolyte	Potential (V vs. RHE)	FE_{CO} (%)	j_{CO} (mA cm^{-2})	Potential window (FE_{CO}>90%)	TOF (h^{-1})	Ref.
**H-3DOM-ZnN_4/P-C**	0.1 M $KHCO_3$	−0.6<br>−0.9	~100<br>90.1	2.9<br>5.7	−0.4~−0.9	7.8×10^4	This work
**ZnN_x/C**	0.5 M $KHCO_3$	−0.43	95	~2	−0.43	9.9×10^3	9
**Ni–N_4–C**	0.1 M $KHCO_3$	−0.81	99	28.6	−0.5~−0.9	/	10
**Ni–N–C**	0.1 M $KHCO_3$	−0.65	96	8.2	−0.6~−0.9	2.3×10^3	11
**Ni–N–C**	0.1 M $KHCO_3$	−1.2	90	12	/	292	12
**Ni–N-Gr**	0.1 M $KHCO_3$	−0.7	95	0.5	−0.7~−0.9	4.6×10^3	13
**Ni@NCH-1000**	0.1 M $KHCO_3$	−1	96	3.8	−0.9~−1.0	/	14
**FeNC**	0.1 M $KHCO_3$	−0.6	85	~5	/	1.0×10^3	15
**Fe-SA-900**	0.1 M $KHCO_3$	−0.58	90	/	−0.58	386	16
**FeNPCN**	0.1 M $KHCO_3$	−0.5	94	0.1	−0.4~−0.5	/	17
**Cu-N_4-C/1100**	0.1 M $KHCO_3$	−0.9	98	~3	−0.6~−1.1	~1.0×10^3	18
**Ag_1–N_3/PCNC**	0.1 M $KHCO_3$	−0.37	95	~4	−0.3~−0.5	9.0×10^3	19
**Sn-C_2O_2F**	0.1 M $KHCO_3$	−0.75	95.2	~8	−0.2~−0.6	1.9×10^3	20

**Cu–Fe–N_6–C**	0.1 M $KHCO_3$	−0.7	98	4	−0.6~−0.9	254	21
**Co–N–Ni/NPCNSs**	0.1 M $KHCO_3$	−0.5	96.4	~8	−0.4~−0.7	2.05×10^3	22

"/" indicates that the information provided in the text is insufficient to obtain the value.

Table S5. The C_{dl} value of different catalysts obtained by EIS test.

Catalysts	C_{dl} ($mF\cdot cm^{-2}$)
H-3DOM-ZnN_4/P-C	28.5
3DOM-ZnN_4/P-C	9.2
3DOM-ZnN_4/C	7.8

Table S6. The Zn content of catalysts obtained at different calcination temperatures.

Temperature (°C)	Zn content (wt%)
850	2.71
950	1.56
1050	0.52

Table S7. The Bader charge and oxidation state of Zn in different unites.

	**Bader charge on Zn (e)**	**Oxidation state (e)**
**ZnN_4**	10.70	+1.29
**ZnN_4/P(II)**	10.73	+1.27
**ZnN_4/P(III)**	10.85	+1.15
**ZnN_4/P(IV)**	10.83	+1.17

Table S8. Comparison of HzOR performances of H-3DOM-ZnN_4/P-C with other reported transition metal-based catalysts.

Catalysts	Electrolyte	N_2H_4 (mM)	E_{onset} (V vs. RHE)	j_{peak} (mA cm^{-2})	Ref
**H-3DOM-ZnN_4/P-C**	1 M KOH	100	0.21	129.8	This work
**Fe-NC-2-1000**	1 M KOH	100	0.28	103.0	23
**wash-Fe_2MoC@NC**	1 M KOH	100	0.28	12.77	24
**SeNCM**	1 M KOH	100	0.34	30.80	25
**MnO/N-C**	1 M KOH	100	0.40	6.28	26
**Fe_2O_3/ECP-15**	1 M KOH	100	0.67	89.57	27
**NSC**	0.1 M KOH	50	0.43	2.92	28
**PolyCuDAB–CB**	0.1 M KOH	10	0.61	11.6	29
**Cu NCs/AC**	0.1 M KOH	100	0.60	20.0	30
**CuO/C**	0.1 M KOH	10	0.98	4.59	31
**S-RGO/Cu**	0.1 M KOH	10	0.98	14.68	32

References:

(1) Kresse, G.; Furthmüller, J. Efficient iterative schemes for ab initio total-energy calculations using a plane-wave basis set. *Phys. Rev. B* **1996**, *54*, 11169-11186.
(2) Kresse, G.; Hafner, J. Ab initio. *Phys. Rev. B* **1994**, *49*, 14251-14269.
(3) Blöchl, P. E. Projector augmented-wave method. *Phys. Rev. B* **1994**, *50*, 17953-17979.
(4) Perdew, J. P.; Burke, K.; Ernzerhof, M. Generalized Gradient Approximation Made Simple. *Phys. Rev. Lett.* **1996**, *77*, 3865-3868.
(5) Zhang, Y.; Yang, W. Comment on "Generalized Gradient Approximation Made Simple". *Phys. Rev. Lett.* **1998**, *80*, 890-890.
(6) Hammer, B.; Hansen, L. B.; Nørskov, J. K. Improved adsorption energetics within density-functional theory using revised Perdew-Burke-Ernzerhof functionals. *Phy. Rev. B* **1999**, *59*, 7413-7421.
(7) Monkhorst, H. J.; Pack, J. D. Special points for Brillouin-zone integrations. *Phy. rev. B* **1976**, *13*,

5188.
(8) Nørskov, J. K.; Rossmeisl, J.; Logadottir, A.; Lindqvist, L.; Kitchin, J. R.; Bligaard, T.; Jonsson, H. Origin of the overpotential for oxygen reduction at a fuel-cell cathode. *J. Phys. Chem. B* **2004**, *108*, 17886-17892.
(9) Yang, F.; Song, P.; Liu, X.; Mei, B.; Xing, W.; Jiang, Z.; Gu, L.; Xu, W. Highly Efficient CO_2 Electroreduction on ZnN_4-based Single-Atom Catalyst. *Angew. Chem. Int. Ed.* **2018**, *57*, 12303-12307.
(10) Li, X.; Bi, W.; Chen, M.; Sun, Y.; Ju, H.; Yan, W.; Zhu, J.; Wu, X.; Chu, W.; Wu, C.; Xie, Y. Exclusive Ni-N4 Sites Realize Near-Unity CO Selectivity for Electrochemical CO_2 Reduction. *J. Am. Chem. Soc.* **2017**, *139*, 14889-14892.
(11) Pan, F.; Deng, W.; Justiniano, C.; Li, Y. Identification of champion transition metals centers in metal and nitrogen-codoped carbon catalysts for CO_2 reduction. *Appl. Catal. B: Environ.* **2018**, *226*, 463-472.
(12) Möller, T.; Ju, W.; Bagger, A.; Wang, X.; Luo, F.; Ngo Thanh, T.; Varela, A. S.; Rossmeisl, J.; Strasser, P. Efficient CO_2 to CO electrolysis on solid Ni–N–C catalysts at industrial current densities. *Energy Environ. Sci.* **2019**, *12*, 640-647.
(13) Su, P.; Iwase, K.; Nakanishi, S.; Hashimoto, K.; Kamiya, K. Nickel-Nitrogen-Modified Graphene: An Efficient Electrocatalyst for the Reduction of Carbon Dioxide to Carbon Monoxide. *Small* **2016**, *12*, 6083-6089.
(14) Daiyan, R.; Zhu, X.; Tong, Z.; Gong, L.; Razmjou, A.; Liu, R.-S.; Xia, Z.; Lu, X.; Dai, L.; Amal, R. Transforming active sites in nickel–nitrogen–carbon catalysts for efficient electrochemical CO_2 reduction to CO. *Nano Energy* **2020**, *78*, 105213.
(15) Wu, S.; Lv, X.; Ping, D.; Zhang, G.; Wang, S.; Wang, H.; Yang, X.; Guo, D.; Fang, S. Highly exposed atomic Fe–N active sites within carbon nanorods towards electrocatalytic reduction of CO2 to CO. *Electrochim. Acta* **2020**, *340*, 135930.
(16) Xu, C.; Vasileff, A.; Wang, D.; Jin, B.; Zheng, Y.; Qiao, S.-Z. Synergistic catalysis between atomically dispersed Fe and a pyrrolic-N-C framework for CO_2 electroreduction. *Nanoscale Horiz.* **2019**, *4*, 1411-1415.
(17) Zhong, H.; Meng, F.; Zhang, Q.; Liu, K.; Zhang, X. Highly efficient and selective CO2 electro-reduction with atomic Fe-C-N hybrid coordination on porous carbon nematosphere. *Nano Res.* **2019**, *12*, 2318-2323.
(18) Cheng, H.; Wu, X.; Li, X.; Nie, X.; Fan, S.; Feng, M.; Fan, Z.; Tan, M.; Chen, Y.; He, G. Construction of atomically dispersed Cu-N4 sites via engineered coordination environment for high-efficient CO_2 electroreduction. *Chem. Eng. J.* **2021**, *407*, 126842.
(19) Sui, R.; Pei, J.; Fang, J.; Zhang, X.; Zhang, Y.; Wei, F.; Chen, W.; Hu, Z.; Hu, S.; Zhu, W.; Zhuang, Z. Engineering Ag-Nx Single-Atom Sites on Porous Concave N-Doped Carbon for Boosting CO_2 Electroreduction. *ACS Appl. Mater. Inter.* **2021**, *13*, 17736-17744.
(20) Ni, W.; Gao, Y.; Lin, Y.; Ma, C.; Guo, X.; Wang, S.; Zhang, S. Nonnitrogen Coordination Environment Steering Electrochemical CO_2-to-CO Conversion over Single-Atom Tin Catalysts in a Wide Potential Window. *ACS Catal.* **2021**, *11*, 5212-5221.
(21) Yun, R.; Zhan, F.; Wang, X.; Zhang, B.; Sheng, T.; Xin, Z.; Mao, J.; Liu, S.; Zheng, B. Design of Binary Cu-Fe Sites Coordinated with Nitrogen Dispersed in the Porous Carbon for Synergistic CO_2 Electroreduction. *Small* **2021**, *17*, 2006951.
(22) Pei, J.; Wang, T.; Sui, R.; Zhang, X.; Zhou, D.; Qin, F.; Zhao, X.; Liu, Q.; Yan, W.; Dong, J.;

Zheng, L.; Li, A.; Mao, J.; Zhu, W.; Chen, W.; Zhuang, Z. N-Bridged Co–N–Ni: new bimetallic sites for promoting electrochemical CO2 reduction. *Energy Environ. Sci.* **2021**, *14*, 3019-3028.
(23) Zheng, Y.; He, F.; Chen, M.; Zhang, J.; Hu, G.; Ma, D.; Guo, J.; Fan, H.; Li, W.; Hu, X. Mimicking Hydrazine Dehydrogenase for Efficient Electrocatalytic Oxidation of N2H4 by Fe-NC. *ACS Appl. Mater. Inter.* **2020**, *12*, 38183-38191.
(24) Kasinath Ojha, E. M. F., Tomer Y. Burshtein, and David Eisenberg A Multi-Doped Electrocatalyst for Efficient Hydrazine Oxidation. *Angew. Chem. Int. Ed.* **2018**, *57*, 17168 -17172.
(25) Wang, T.; Wang, Q.; Wang, Y.; Da, Y.; Zhou, W.; Shao, Y.; Li, D.; Zhan, S.; Yuan, J.; Wang, H. Atomically Dispersed Semimetallic Selenium on Porous Carbon Membrane as an Electrode for Hydrazine Fuel Cells. *Angew. Chem. Int. Ed.* **2019**, *58*, 13466-13471.
(26) Ding, J.; Kannan, P.; Wang, P.; Ji, S.; Wang, H.; Liu, Q.; Gai, H.; Liu, F.; Wang, R. Synthesis of nitrogen-doped MnO/carbon network as an advanced catalyst for direct hydrazine fuel cells. *J. Power Sources* **2019**, *413*, 209-215.
(27) Wang, Y.; Chen, Z.; Wu, H.; Xiao, F.; Cao, E.; Du, S.; Wu, Y.; Ren, Z. Self-Assembly-Induced Mosslike Fe_2O_3 and FeP on Electro-oxidized Carbon Paper for Low-Voltage-Driven Hydrogen Production Plus Hydrazine Degradation. *ACS Sustain. Chem. Eng.* **2018**, *6*, 15727-15736.
(28) Cazetta, A. L.; Zhang, T.; Silva, T. L.; Almeida, V. C.; Asefa, T. Bone char-derived metal-free N- and S-co-doped nanoporous carbon and its efficient electrocatalytic activity for hydrazine oxidation. *Appl. Catal. B: Environ.* **2018**, *225*, 30-39.
(29) He, F.; Mi, L.; Shen, Y.; Chen, X.; Yang, Y.; Mei, H.; Liu, S.; Mori, T.; Zhang, Y. Driving electrochemical oxygen reduction and hydrazine oxidation reaction by enzyme-inspired polymeric Cu(3,3′-diaminobenzidine) catalyst. *J. Mater. Chem. A* **2017**, *5*, 17413-17420.
(30) Gao, X.; Du, C.; Zhang, C.; Chen, W. Copper Nanoclusters on Carbon Supports for the Electrochemical Oxidation and Detection of Hydrazine. *ChemElectroChem* **2016**, *3*, 1266-1272.
(31) Ma, Y.; Li, H.; Wang, R.; Wang, H.; Lv, W.; Ji, S. Ultrathin willow-like CuO nanoflakes as an efficient catalyst for electro-oxidation of hydrazine. *J. Power Sources* **2015**, *289*, 22-25.
(32) Liu, C.; Zhang, H.; Tang, Y.; Luo, S. Controllable growth of graphene/Cu composite and its nanoarchitecture-dependent electrocatalytic activity to hydrazine oxidation. *J. Mater. Chem. A* **2014**, *2*, 4580-4587.
